# The transcriptional repressor Ssn6 modulates phase separation to regulate fungal gene expression

**DOI:** 10.1128/mbio.00316-26

**Published:** 2026-03-30

**Authors:** Maureen A. Dowell, Mae I. Staples, Corey Frazer, Richard J. Bennett

**Affiliations:** 1 Department of Molecular Microbiology and Immunology, Brown University 6752, Providence, Rhode Island, USA; Instituto Carlos Chagas, Curitiba, Brazil

**Keywords:** transcription, corepressors, phase separation, epigenetic

## Abstract

The transcriptional regulation of cell fate plays a central role in eukaryotic cell differentiation. In the human fungal pathogen *Candida albicans*, the white-to-opaque cell fate switch is controlled by an interconnected network of eight transcription factors (TFs). These include Ssn6, a negative regulator of the opaque state that can function with its co-repressor Tup1; together, they form a global repressor complex that is conserved from yeast to mammals. Here, we evaluated the roles of four Ssn6 domains (N, TPR, M, and CTD) in white-opaque switching. Loss of the prion-like N or M domains had limited effects on Ssn6 phenotypes. In contrast, the tetratricopeptide repeat (TPR) domain was critical for function, consistent with this domain mediating interactions with Tup1 and DNA-binding TFs. The intrinsically disordered C-terminal domain (CTD) showed complex roles; deletion of this domain increased Ssn6 activity whereas substitution of acidic residues within this region abolished Ssn6 function. Notably, these phenotypes were linked to the phase separation capacity of Ssn6, as changes to the TPR or CTD altered the properties of Ssn6 condensates in human U2OS cells. Experiments using purified Ssn6 (± Tup1) demonstrated that this protein is recruited into condensates formed by other white-opaque-regulating TFs and alters condensate properties in a TPR- and CTD-dependent manner. Together, these experiments reveal how individual Ssn6 domains can modulate the phase separation properties of DNA-binding TFs and thereby regulate gene expression and cell fate.

**IMPORTANCE:**

The mechanisms by which transcription factors (TFs) and co-regulators control gene expression remain ill-defined. An interconnected network of TFs regulates an epigenetic switch between white and opaque states in *Candida albicans* and serves as a model to understand the transcriptional regulation of eukaryotic cell fate. Here, we examine the role of Ssn6, one of the eight core regulators of the white-opaque switch, and reveal key roles for both the structured TPR domain and the disordered C-terminal domain. We demonstrate that Ssn6 is readily incorporated into transcriptional condensates where it disrupts the liquid-like properties of these condensates, both in the presence and absence of the co-repressor Tup1. Together with studies in higher eukaryotes, these results suggest a conserved role for TPR-containing proteins in regulating gene expression via the modulation of the physical properties of transcriptional condensates.

## INTRODUCTION

Transcriptional regulation plays a central role in eukaryotic cell differentiation including in the fungal pathobiont *Candida albicans*. This species is a frequent commensal in the human gastrointestinal and urogenital tracts but can also cause mucosal and systemic disease, with its ability to adapt to different environments enabled by its morphological plasticity (1). This is exemplified by a heritable and reversible transition between “white” and “opaque” states that show differences in their metabolism, tissue tropism, virulence, and mating competence (1, 2). The regulation of the white-opaque switch involves reprogramming of >1,000 genes and shows close mechanistic parallels to the transcriptional control of cell differentiation in higher eukaryotes (3).

At its core, the white-opaque switch is regulated by an integrated network of eight master transcription factors (TFs), each of which binds to its own promoter and to the promoters of the other TFs in the network (4–6). Several of these TFs have been shown to form condensates by undergoing liquid-liquid phase separation, which may enable co-assembly into transcriptional complexes (7–9). The white-opaque TF network includes the transcriptional repressor Ssn6, with loss of this gene locking cells in the opaque state (6). Ssn6 lacks DNA-binding capacity and is recruited to promoters via interactions with sequence-specific DNA-binding TFs.

In the model yeast *Saccharomyces cerevisiae*, Ssn6 (Cyc8) has been well-characterized as a global repressor together with its partner Tup1 (10–12). *S. cerevisiae* Ssn6 contains 10 tetratricopeptide repeats (TPRs), of which the first three repeats mediate interactions with the N-terminal domain of Tup1 (13). Ssn6 can also interact with a wide variety of DNA-binding TFs, and this flexibility allows control over genes involved in glucose repression, oxidative stress, and cell type specificity (10, 11, 14–16). Functional homologs of Ssn6 and Tup1 exist in higher eukaryotes, including UTX/UTY and the Groucho/TLE family, respectively (16–19). *S. cerevisiae* Ssn6 can interact with TLE family members and mediate transcriptional repression when transfected into mammalian cells, demonstrating conservation of function (16, 17).

Despite extensive studies into Ssn6/Tup1, the regulatory mechanisms of these proteins remain only partially defined. Current models suggest that Ssn6−Tup1 generates a repressive state via the recruitment of histone deacetylases (HDACs) or by changes to nucleosome positioning (16, 20–23). In addition, this complex may block transcription by physically interfering with the core transcriptional machinery (16, 24–26). Tup1 has generally been implicated in gene repression while Ssn6 acts to recruit Tup1 to promoters via interactions with DNA-binding proteins (16). Other studies have established that Ssn6−Tup1 can also function as an activator of transcription at certain *S. cerevisiae* genes (27–29). While Ssn6 and Tup1 often function together, their roles are not entirely overlapping, with Ssn6 regulating the repression of a broader set of genes than Tup1 (30). These studies indicate that the functions of Ssn6 and Tup1 are complex and context dependent.

In contrast to *S. cerevisiae*, few studies have examined Ssn6 function in *C. albicans*. Initial reports indicated a role for Ssn6 (and Tup1) in repressing filamentous growth in *C. albicans* (31, 32), highlighting parallels with *S. cerevisiae* (16). *C. albicans* Ssn6 was also identified as a virulence factor with both overexpression and deletion of *SSN6* leading to a defect in systemic infection (31). Clear differences have been observed between *C. albicans ssn6Δ/Δ* and *tup1Δ*/*Δ* phenotypes indicating that these genes perform distinct functions, and Ssn6 represses a subset of genes even in the absence of Tup1 (11, 31). However, the mechanisms by which *C. albicans* Ssn6 interacts with other TFs, co-regulates gene expression with Tup1, and represses the white-to-opaque switch remain undefined.

Here, we address the role of each of the four Ssn6 domains (N, TPR, M, and CTD) and show that both the TPR and CTD are critical for Ssn6 function in white-opaque switching. Notably, Ssn6 forms phase-separated condensates in the nuclei of mammalian cells, and condensate size increased when co-expressed with Tup1. Purified Ssn6 (± Tup1) is also readily incorporated into condensates formed by other white-opaque TFs. This incorporation alters the viscoelastic properties of TF condensates, leading to decreased liquid-like properties and condensate disruption. Together, this work dissects the functions of individual Ssn6 domains and links Ssn6 function to the modification of transcriptional condensates.

## RESULTS

### Ssn6 TPR and CTD domains affect white-opaque switching

**Fig 1:**
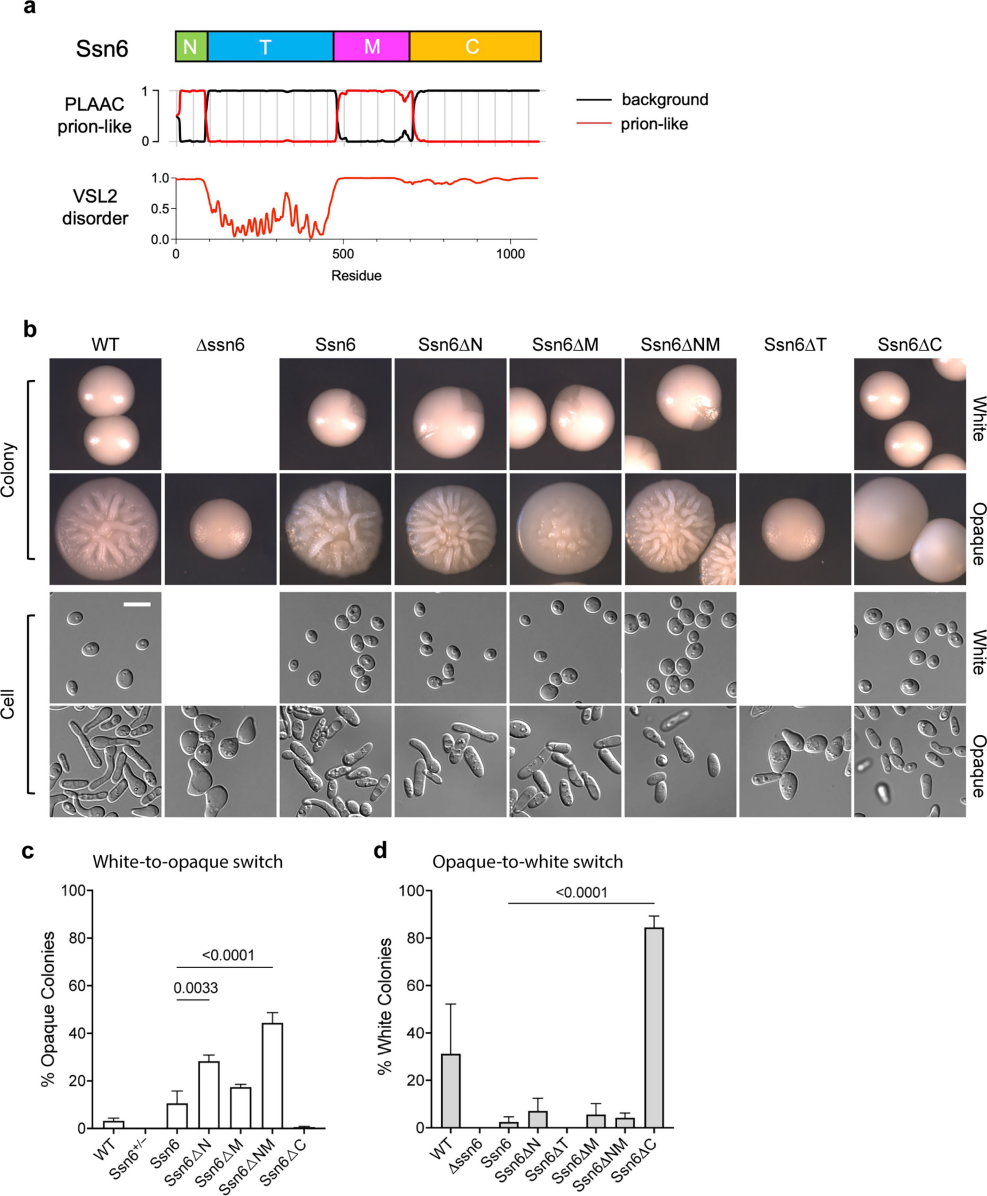
Analysis of Ssn6 domain deletion mutants in white-opaque switching assays. (**a**) Diagram of Ssn6 domains: N-terminal prion-like domain (N, residues 1–86), tetratricopeptide repeat (T, residues 87–478), medial prion-like domain (M, residues 479–706), and disordered C-terminus (C, residues 707–1080). (**b**) Images of *Candida albicans* cells expressing Ssn6 variants. Top section shows colony morphology. White colonies include those both with and without opaque (darker) sectors. Bottom row shows DIC images of cells. Scale 10 µm. (**c**) Frequency of white Ssn6 variant-expressing cells switching to opaque. (**d**) Frequency of opaque Ssn6 variant-expressing cells switching to white. Statistics shown for Fisher’s least significant difference (LSD) test versus Ssn6 full-length, *P* ≤ 0.05.

*C. albicans* Ssn6 is a 1080 amino acid protein that contains nine tetratricopeptide repeats (TPRs) that are implicated in interactions with Tup1 and sequence-specific DNA-binding TFs (10, 11, 13, 16). Prion-like amino acid composition analysis previously identified two prion-like domains (PrLDs) in Ssn6 (7, 33). Analysis of intrinsically disordered regions by VSL2 (34) showed that the C-terminal domain (CTD) is disordered but not prion-like (Fig. 1a). For this work, Ssn6 is therefore defined as having four domains: an N-terminal PrLD (N), a structured TPR domain (TPR or T), a medial PrLD (M), and a disordered CTD (Fig. 1a).

Ssn6 is one of eight TFs that form an interconnected network regulating white-opaque switching in *C. albicans* (6). This network includes both positive and negative regulators of the opaque state, with deletion of Ssn6 causing switching to the opaque state *en masse* (6). Tup1, considered a co-repressor with Ssn6, also acts to repress opaque-specific genes (35). To investigate the role of Ssn6 in the regulation of switching, Ssn6 variants lacking each of the four individual domains were integrated at the endogenous *SSN6* locus in a *ssn6Δ/Δ* strain (36). A full-length *SSN6* re-integrant was used as a control. Expression levels of wild-type (WT) and Ssn6 variants were similar (~2-fold differences observed; Fig. S1b), and all variants correctly localized to the nucleus (Fig. S1a).

To quantify white-opaque switching, pure populations of white and opaque cells were plated for single colonies on synthetic complete dextrose (SCD) medium and incubated for 7 days at 22°C, after which colonies were scored for switching. Deletion of *SSN6* resulted in the formation of colonies locked in an opaque-like state in line with a previous study (6), although cell morphologies were aberrant and did not closely resemble WT white or opaque cells (Fig. 1b and c). Expression of all Ssn6 variants supported formation of the white cell state except for Ssn6∆T which formed cells similar to *ssn6Δ/Δ* cells (Fig. 1b and c), indicating that the TPR is essential for Ssn6 function. Loss of either the N or M PrLDs had only a modest effect on switching; both mutants exhibited elevated rates of white-to-opaque switching compared to the control, which further increased in a mutant lacking both PrLDs (Ssn6ΔNM; Fig. 1c). Interestingly, Ssn6∆C strains lacking the CTD showed very low white-to-opaque switching frequencies under these conditions (Fig. 1c) together with an increased frequency of reverse opaque-to-white switching (Fig. 1d). Taken together, these experiments reveal that (i) the TPR domain is essential for Ssn6’s ability to support the white state, (ii) the N and M PrLDs have only a modulatory role in cell type switching (contrasting with the essential roles of PrLDs in the function of opaque-promoting TFs [7]), and (iii) removal of the unstructured CTD results in a hyperactive Ssn6 protein that biases cells toward forming the white state.

### Ssn6 domains make distinct contributions to white and opaque gene expression profiles

**Fig 2:**
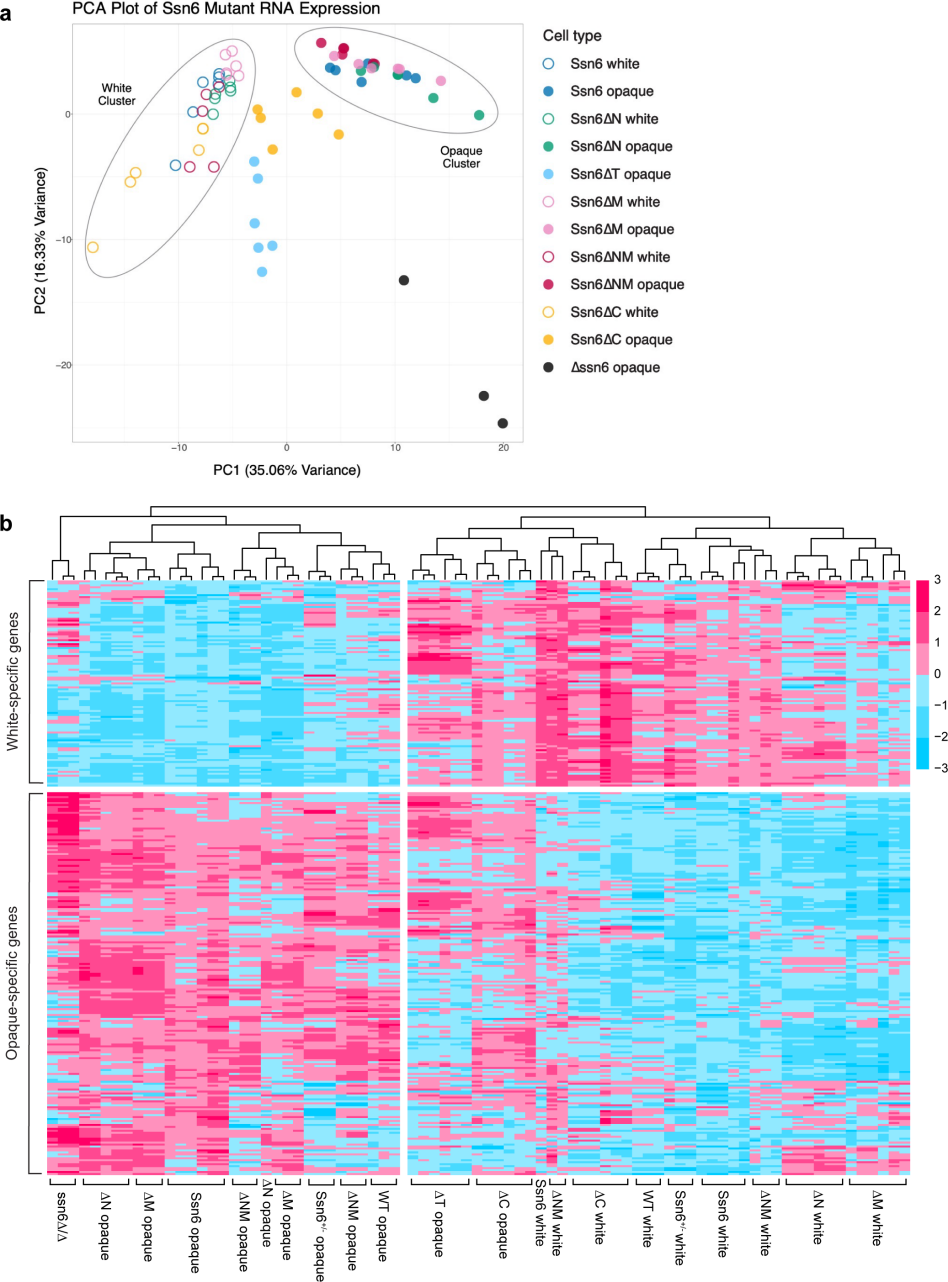
RNA expression profiling of Ssn6 variant expressing cells in white and opaque states. (**a**) Principal component analysis of RNA-sequencing data for strains expressing different Ssn6 variants and grown in SCD medium. (**b**) Heatmap showing differentially expressed genes between white and opaque strains expressing different Ssn6 variants. Scale represents *z*-score.

White and opaque cells show extensive differences in gene expression with >1,000 genes (out of ~6,100) differentially expressed between the two states (3). RNA sequencing was performed on Ssn6 variant-expressing cells in both white and opaque states for a total of 81 samples. Principal component analysis (PCA) (Fig. 2a) and heatmaps of differentially expressed genes (Fig. 2b) were used to compare gene expression profiles between strains.

RNA profiles generally grouped into two main PCA clusters that represented white and opaque cell states. Analysis of PrLD deletions (∆N, ∆M, and ∆NM) showed that these cells exhibited expression profiles similar to those of control white/opaque cells (Fig. 2a and b). This is in line with the modest effects seen upon deleting N or M domains on white-opaque switching frequencies (Fig. 1). Notably, the expression profile of *ssn6Δ/Δ* cells, although forming darker, opaque-like colonies, did not cluster with that of control opaque cells (Fig. 2a). In particular, many white-enriched genes were expressed at a higher level in this mutant (elevated genes shown in red; Fig. 2b) than in control opaque cells. This is consistent with Ssn6 reducing the expression of a number of white-enriched genes in opaque cells (6).

Ssn6∆T cells exhibited cell and colony phenotypes similar to *ssn6Δ/Δ* opaque-like cells, consistent with the TPR domain being critical for Ssn6 function (Fig. 1b). Despite these phenotypic similarities, the expression profile of Ssn6∆T cells was clearly distinct from that of both *ssn6Δ/Δ* and WT opaque cells (Fig. 2a and b). In fact, loss of the TPR resulted in a profile that clustered more closely to that of WT white cells than to WT opaque cells. This clustering was due to high expression of numerous white-enriched genes in Ssn6∆T “opaque” cells, as well as the reduced expression of many genes that are normally elevated in opaque cells (Fig. 2b). Loss of the TPR domain therefore results in “opaque” cells that are distinct from the “opaque” cells formed by *ssn6Δ/Δ* mutants.

Interestingly, loss of the CTD also had complex consequences for gene expression. The profile of Ssn6∆C “opaque” cells most closely resembled that of Ssn6∆T “opaque” cells, while the profile of Ssn6∆C white cells also clustered away from most other white cell profiles (Fig. 2a and b). Ssn6∆C “opaque” cells showed high expression of both white- and opaque-enriched genes similar to Ssn6∆T “opaque” cells (Fig. 2B), again indicating a general lack of repression of white-enriched genes in this state. These results indicate that loss of the CTD has a large impact on gene expression, particularly in the opaque state where the profile is closest to cells lacking the TPR domain.

Together, these experiments highlight the distinct roles of individual Ssn6 domains in regulating white-opaque gene expression. They establish that (i) *ssn6Δ/Δ* “opaque” cells express a different gene set from control opaque cells, (ii) loss of the TPR does not phenocopy loss of the whole *SSN6* gene indicative of TPR-independent functions, and (iii) loss of the CTD results in “opaque” cells with a profile closest to that of Ssn6∆T “opaque” cells and that these cells are again distinct from control opaque cells.

### Ssn6 forms dynamic condensates when expressed in mammalian cells

The PrLDs of several white-opaque-regulating TFs form phase-separated condensates when expressed in the nuclei of mammalian U2OS cells (7, 8). We therefore evaluated the potential for Ssn6 (or sub-domains of this protein) to form condensates using U2OS cells harboring an array of ~50,000 copies of the LacO sequence (37, 38). Ssn6 variants were expressed as fusion proteins with eYFP-LacI; LacI is recruited to the LacO array thereby concentrating LacI fusion proteins to a focal point in the nucleus of these cells (7, 8).

**Fig 3:**
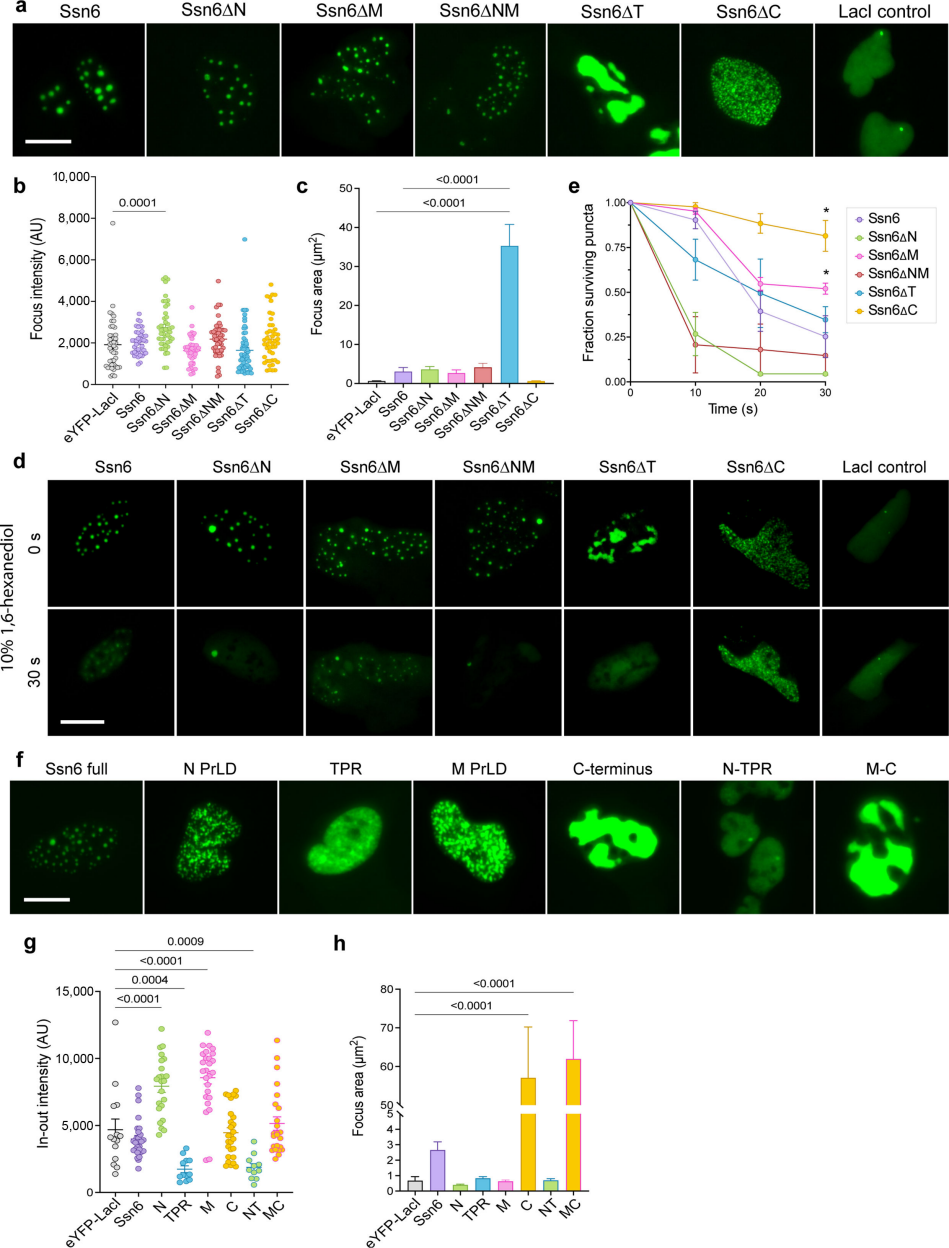
Ssn6 forms phase-separated condensates in U2OS cells. (**a**) eYFP-Ssn6-LacI plasmids expressed in U2OS-LacO cells. Different Ssn6 variants are expressed as shown. (**b**) Quantification of condensate area in eYFP-Ssn6-LacI U2OS condensates. (**c**) Quantification of average fluorescence intensity of eYFP-Ssn6-LacI foci in U2OS cells compared to those expressing the control eYFP-LacI vector. (**d**) Images from a time lapse of 10% 1,6-hexanediol treatment on U2OS-LacO cells expressing different eYFP-Ssn6-LacI constructs. (**e**) Quantification of surviving puncta following hexanediol treatment in d. Comparisons shown against Ssn6 at 30 s. (**f**) Expression of Ssn6 single domains in U2OS cells. (**g**) Quantification of average fluorescence intensity of Ssn6 single domain condensates in U2OS cells in panel f. (**h**) Quantification of condensate area in Ssn6 single domain condensates in U2OS cells shown in panel f. Statistics were computed for Fisher’s LSD test against eYFP-LacI (g and h) or eYFP-LacI and Ssn6 (b and c); *P*-values ≤ 0.05 are shown. Scale bars, 10 µm.

Full-length Ssn6 formed large spherical foci at multiple positions in U2OS nuclei, whereas the LacI control formed smaller puncta only at the LacO array (Fig. 3a through c). Ssn6 mutants lacking PrLDs (Ssn6ΔN, Ssn6ΔM, and Ssn6ΔNM) also formed large condensates using this system (Fig. 3a through c). In contrast, expression of the Ssn6∆T variant resulted in large amorphous structures (“blobs”) that filled much of the nucleus; these structures were substantially larger than foci formed by LacI or full-length Ssn6 (Fig. 3a through c). Expression of the Ssn6∆C variant produced only small, granular-looking puncta suggesting that deletion of the CTD reduces dynamic interactions by Ssn6 (Fig. 3a through c).

To test for liquid-like behavior, cells were treated with 10% 1,6-hexanediol, a chemical that can dissolve liquid condensates (38–41). Full-length Ssn6 puncta rapidly reduced in size/quantity within 30 s of 1,6-hexanediol addition (Fig. 3d and e). Puncta formed by Ssn6∆N and Ssn6∆NM also quickly dissolved, although Ssn6∆M puncta only partially dissipated (Fig. 3d and e). The amorphous structures formed by Ssn6∆T also dissolved upon hexanediol treatment (Fig. 3d and e). Notably, hexanediol did not dissolve Ssn6∆C puncta, further establishing that the CTD is important for the liquid-like behavior of Ssn6 condensates (Fig. 3d and e).

We also examined the properties of individual Ssn6 domains fused to eYFP and LacI in the U2OS-LacO system (Fig. 3f). Expression of the TPR domain did not produce discrete puncta, while the CTD produced amorphous structures similar to those formed by Ssn6∆T (Fig. 3f). The N and M PrLDs formed small, granular puncta that were significantly brighter than the LacI control (Fig. 3f and g). We also expressed the two halves of Ssn6 in U2OS cells; expression of the N and TPR domains (N-TPR) fused to eYFP-LacI produced singular puncta that resembled the LacI control (Fig. 3f through h). Expression of the M and CTD domains (M-C) again produced large blobs that were significantly larger than LacI control puncta, similar to the CTD and Ssn6∆T constructs (Fig. 3d through h). These assays establish that Ssn6 forms liquid-like condensates in U2OS nuclei and that the TPR and CTD domains both have a dominant effect in determining condensate properties.

### Amino acid substitutions to the CTD alter condensate formation and white-opaque switching

Given that loss of the disordered CTD from Ssn6 had a major impact on both white-opaque switching and condensate formation, we examined the effect of amino acid substitutions to this domain on Ssn6 function. Approximately 20% of the CTD is composed of acidic residues (Fig. S2a), and a mutant variant was therefore created where all of these residues within the CTD were mutated to alanine. Several phosphorylation sites have been identified in the CTD (42), and these were mutated to alanine or aspartate to block or mimic phosphorylation, respectively. Lastly, all aromatic residues (Y/F) within the CTD were mutated (to S or A), as aromatic residues are closely associated with the ability of intrinsically disordered regions (IDRs) to form condensates (43, 44).

Each CTD variant was expressed in U2OS cells to evaluate condensate behavior (Fig. S2b through d) and in *C. albicans* cells to determine white-opaque switching capacity (Fig. S2e through g). Interestingly, cells expressing the CTD variant lacking acidic residues were completely blocked from forming the white state, indicating that this variant was non-functional (Fig. S2g). Moreover, in U2OS cells, this mutant generated only small, grainy puncta demonstrating altered condensate properties (Fig. S2b through d). In contrast, Ssn6 variants harboring other amino acid substitutions formed U2OS foci similar to the WT control, although these variants showed increased switching from opaque to white (Fig. S2b through g). These experiments highlight that acidic residues in the CTD, but not other amino acid residues tested, are critical to Ssn6 condensate properties and to the regulation of white-opaque switching.

### Tup1 co-localizes with Ssn6 and increases condensate size in U2OS cells

**Fig 4:**
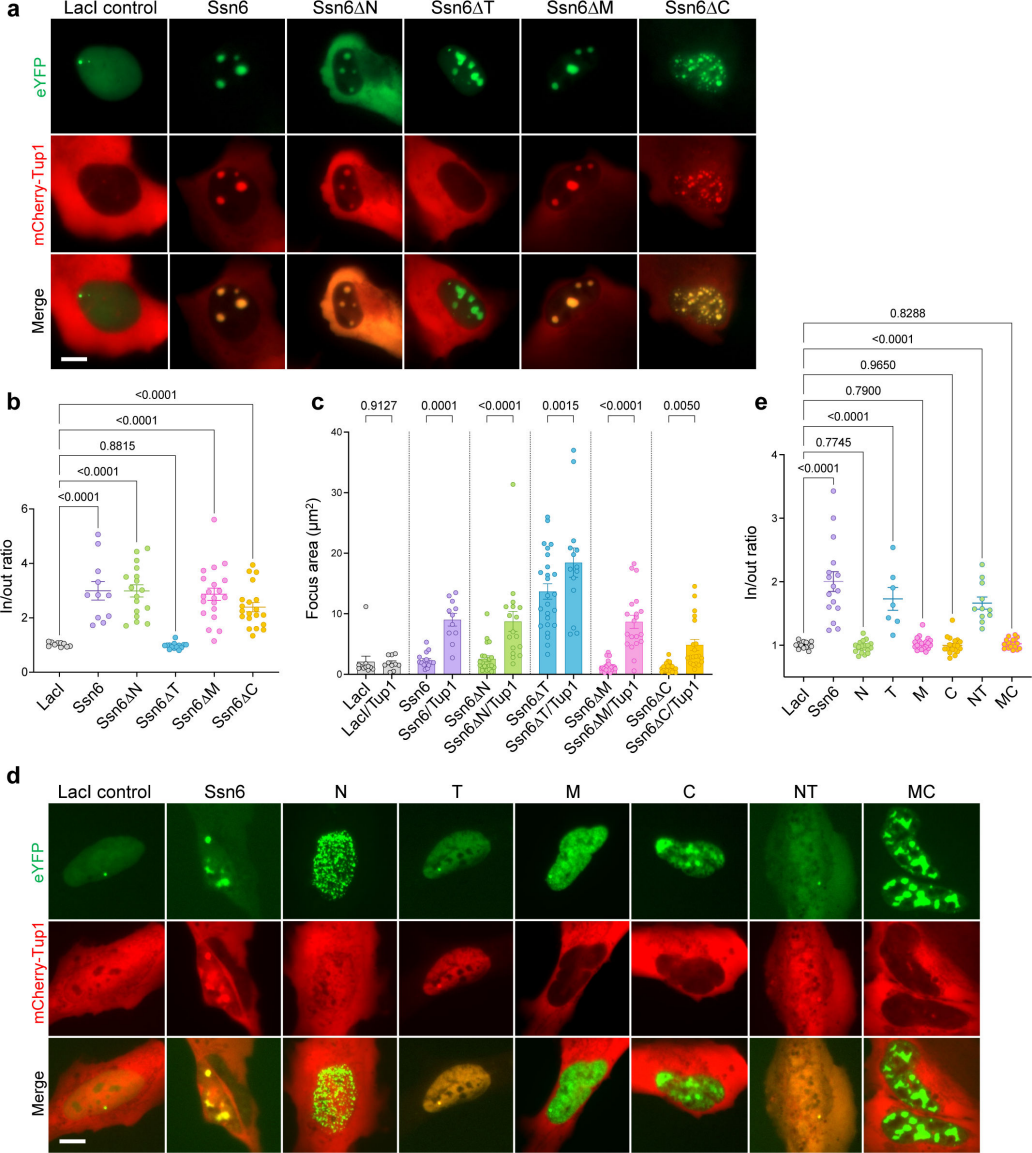
Co-localization of Ssn6 and Tup1 in U2OS cells is dependent on the Ssn6 tetratricopeptide repeat (TPR) domain. (**a**) eYFP-Ssn6-LacI constructs co-transfected into U2OS-LacO cells with mCherry-Tup1. (**b**) Quantification of eYFP and mCherry co-localization in panel a. (**c**) Comparison of eYFP focus size when eYFP-Ssn6-LacI is expressed alone versus co-expression with mCherry-Tup1. (**d**) Individual Ssn6 domains in eYFP-LacI plasmids co-transfected with mCherry-Tup1. (**e**) Quantification of Ssn6 and Tup1 co-localization in panel d. Statistics are shown for Fisher’s LSD test. Scale bars, 10 µm.

Ssn6 often functions with its co-repressor Tup1 (10, 13, 15), and we therefore evaluated the effect of co-expression of Tup1 on Ssn6 condensates. Tup1 was expressed as a fusion to mCherry and co-expressed with eYFP-Ssn6-LacI in the U2OS cell model (Fig. 4a). Notably, Tup1 co-localized with Ssn6 and with all Ssn6 domain variants except for Ssn6∆T (Fig. 4b), consistent with *S. cerevisiae* studies indicating that the TPR domain mediates interactions with Tup1 (13, 45). Interestingly, the presence of Tup1 substantially increased the size of Ssn6 condensates, with Ssn6 foci ~5 times larger when Tup1 was present (compare Ssn6/Tup1 with Ssn6 alone; Fig. 4c). We also co-expressed Tup1 with individual Ssn6 domains, which showed that Tup1 co-localized with the TPR domain (T) but not with other Ssn6 domains (Fig. 4d and e). These experiments establish that Ssn6 interacts with Tup1 via its TPR domain and that Ssn6−Tup1 condensates in U2OS cells are significantly larger than those formed by Ssn6 alone.

### Purified Ssn6−Tup1 alters TF condensate properties

Previous studies showed that white-opaque regulators Efg1, Czf1, Flo8, Wor1, and Wor4 can undergo phase separation *in vitro* (7, 8). Ssn6 was therefore similarly overexpressed and purified from *Escherichia coli* as a fusion to maltose-binding protein (MBP, Fig. S3a and b). Release of Ssn6 from MBP by TEV protease did not readily produce condensates, although aggregated structures formed when polyethylene glycol (PEG) concentrations were present at 7.5% or higher (Fig. S3c). To determine if Tup1 alters the propensity of Ssn6 to phase separate *in vitro*, we co-expressed MBP−Ssn6 and MBP−Tup1 and purified the resulting Ssn6−Tup1 complex (Fig. S3b). This complex also did not form condensates but again formed aggregates with PEG concentrations of 7.5% or above (Fig. S3d and e).

**Fig 5:**
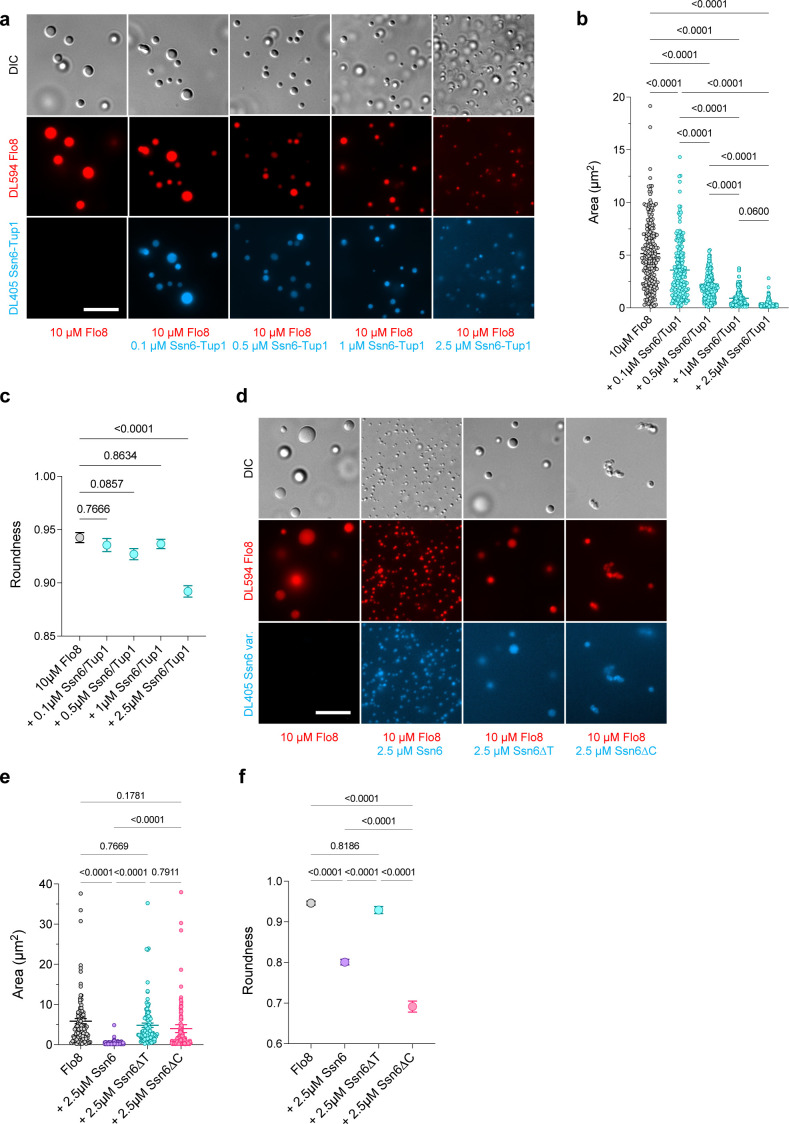
Ssn6 is incorporated into TF condensates *in vitro* and alters their viscoelastic properties. (**a**) Titration of 10 µM MBP−Flo8 with varying concentrations of MBP−Ssn6/MBP−Tup1 and 5% PEG. (**b**) Quantification of Flo8 condensate area in panel a. Statistics in this figure show Tukey’s multiple comparisons test, all comparisons shown unless noted. (**c**) Quantification of Flo8 condensate roundness in panel a. Statistics shown against Flo8 control. (**d**) 10 µM MBP−Flo8 with 2.5 µM MBP−Ssn6, MBP−Ssn6∆T, or MBP−Ssn6∆C in 5% PEG. (**e**) Quantification of Flo8 condensate area in panel d. (**f**) Quantification of Flo8 condensate roundness in panel d. Scale bars 10 µm.

Ssn6 is recruited to promoters via interactions with other TFs (6), and we therefore examined the effect of Ssn6 (± Tup1) on condensates formed by Flo8, a TF that promotes white-to-opaque switching (46–48) and that readily forms condensates *in vitro* (7, 8). In line with previous results, Flo8 at a concentration of 10 µM formed liquid-like droplets in the presence of 5% PEG (8), and yet inclusion of low amounts of Ssn6−Tup1 (0.1 µM) significantly decreased the size of Flo8 condensates (Fig. 5a and b). Moreover, inclusion of 2.5 µM Ssn6−Tup1 significantly decreased both the size and “roundness” of Flo8 condensates (Fig. 5c). Addition of Ssn6 alone (i.e., without Tup1) also resulted in similar decreases in the size and roundness of condensates (Fig. 5d through f).

To determine if the TPR or CTD contributes to the effect of Ssn6 on Flo8 condensates, we purified Ssn6 variants lacking each of these domains from *E. coli* (Fig. S3b). We found that Ssn6∆T was still recruited into Flo8 condensates but did not change the size or roundness of condensates (Fig. 5d though f). This result is notable as it indicates that Ssn6 can interact with Flo8 independent of the TPR domain. Addition of the Ssn6∆C variant to Flo8 condensates decreased the roundness of condensates and resulted in more aggregated structures, although it did not reduce condensate size to the same extent as full-length Ssn6 (Fig. 5d through f). Together, these results reveal that even small relative quantities of Ssn6 (± Tup1) can drastically alter TF condensate properties, and that these changes are impacted by both the TPR and CTD.

## DISCUSSION

In this study, we examine how the global repressor Ssn6 regulates gene expression and cell fate. Ssn6, either individually or with its co-repressor Tup1, impacts the expression of a large fraction of the transcriptome in both *S. cerevisiae* and *C. albicans* (11, 16). Given that homologs of Ssn6 exist in multiple species including humans and fruit flies (16, 17, 49, 50), with links to tissue-specific developmental processes (51–53), this study has broad implications for understanding how a global repressor can control cell fate from yeast to humans.

### Functional dissection of Ssn6

The roles of the four domains of *C. albicans* Ssn6 (N, TPR, M, and CTD) were evaluated in white-opaque switching. In *S. cerevisiae*, the TPR domain mediates interactions with multiple partners including the co-repressor Tup1 (13, 14, 54). We found that the TPR domain was similarly critical for Ssn6 function in *C. albicans*, with loss of this domain causing cells to adopt an opaque-like phenotype similar to that of *ssn6Δ/Δ* cells. We note that cells in these “opaque” states were distinct from those of control opaque cells, with aberrant cellular morphologies evident for both *ssn6Δ/Δ* and Ssn6∆T cells compared to WT opaque cells (Fig. 1b). Surprisingly, the expression profile of Ssn6∆T “opaque” cells was distinct from that of *ssn6Δ/Δ* “opaque” cells (Fig. 2a and b), indicating that Ssn6 can impact expression of a subset of genes through its disordered N, M, and C-terminal domains.

The CTD was found to play an unexpectedly prominent role in *C. albicans* Ssn6 function as loss of this domain resulted in a hyperactive opaque-to-white switching phenotype. The expression profile of Ssn6∆C “opaque” cells was closest to that of Ssn6∆T “opaque” cells, with dysregulation of both white- and opaque-specific genes. Even more strikingly, substitution of acidic CTD residues locked cells in the opaque state indicative of a loss of Ssn6 function. The importance of the *C. albicans* CTD contrasts with *S. cerevisiae* where the C-terminal 150 residues of Ssn6 were deemed dispensable for function (13). Unlike the TPR and CTD, deletion of the N and M PrLDs of *C. albicans* Ssn6 did not have a major impact on function, indicating that these domains play relatively modest roles in switching, at least under the experimental conditions evaluated here.

### Phase separation of Ssn6 and incorporation into TF condensates

*C. albicans* Ssn6 readily formed liquid-like condensates in human U2OS cells similar to other TFs from the core white-opaque network (7), and Ssn6 condensates were significantly larger when co-expressed with Tup1. Removal of the TPR domain blocked Tup1 recruitment into Ssn6 condensates, consistent with the importance of this domain for Ssn6−Tup1 interactions (14, 15). In contrast to the results in U2OS cells, purified Ssn6 protein showed little propensity toward condensate formation *in vitro*, either individually or with Tup1. The difference between *in vitro* and *in vivo* results may be due to Ssn6 recruitment into condensates formed by other TFs in U2OS cells (as further discussed below). Post-translational modifications (PTMs) could also contribute to these differences as phase separation propensities are highly sensitive to PTMs (55–58). Analysis of individual Ssn6 domains in U2OS cells showed that N and M PrLDs formed small, bright puncta, the disordered CTD formed large amorphous “blobs,” and the TPR domain was incapable of forming condensates. Notably, substitution of acidic residues within the CTD inhibited Ssn6 phase separation as well as blocking its function in white-opaque switching, indicating that negatively charged residues in the CTD are essential to both processes.

**Fig 6:**
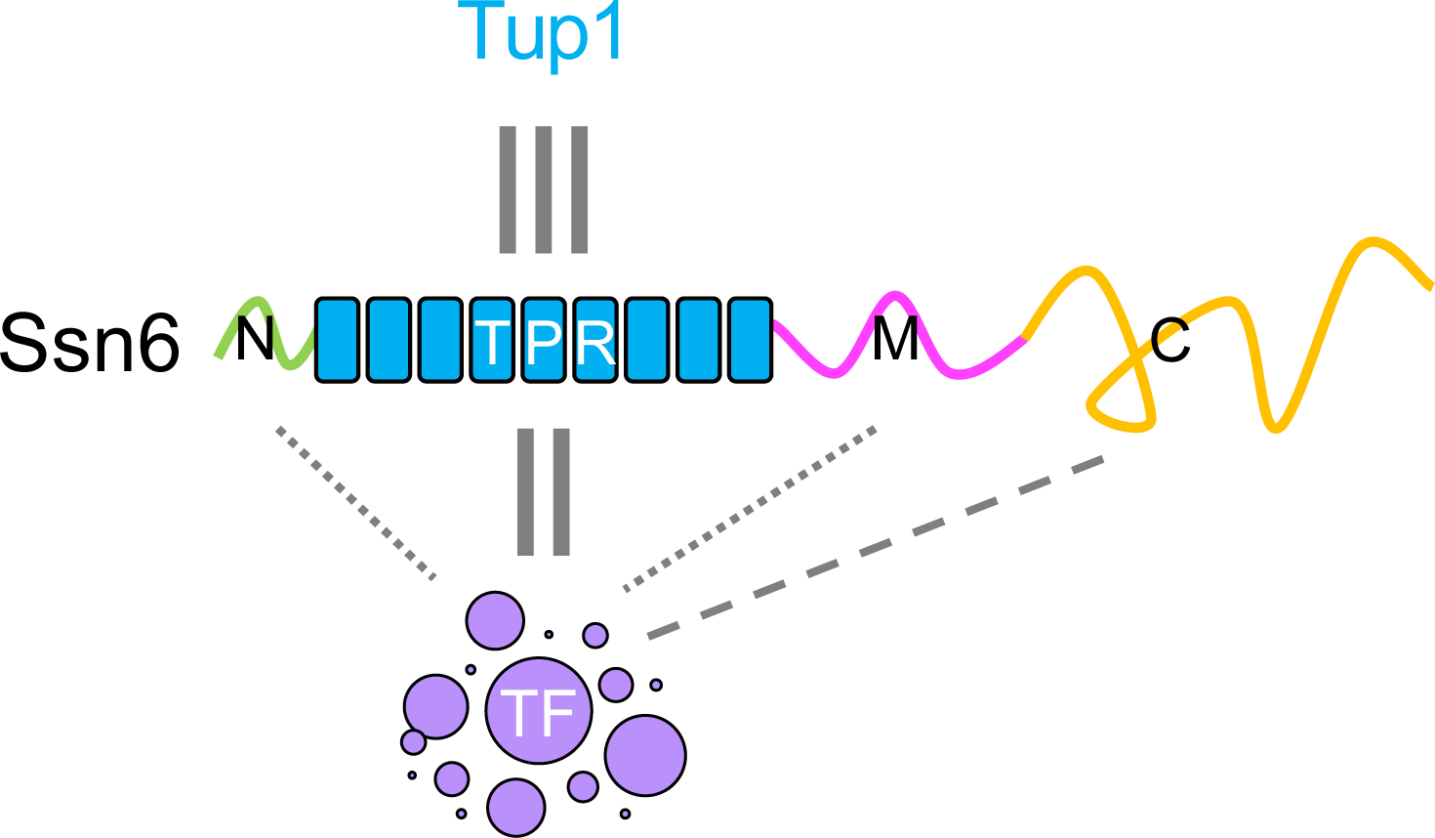
A phase separation model for Ssn6 function. Ssn6 interacts with the co-repressor Tup1 via its TPR domain. Ssn6 is also recruited into liquid condensates formed by other TFs and modifies their biophysical properties. Each Ssn6 domain makes distinct contributions to TF condensate properties, with the TPR and CTD regions particularly important for condensate behavior, whereas the N and M prion-like domains play relatively minor roles in modulating phase separation under the conditions evaluated here.

In contrast to Ssn6 behavior in U2OS cells, purified Ssn6 protein (± Tup1) did not form liquid-like condensates *in vitro* but readily formed aggregates at PEG concentrations of 7.5% or higher. Notably, however, Ssn6 was readily incorporated into liquid condensates formed by the white-opaque regulator Flo8. This TF was previously shown to form condensates, and this process was linked to the assembly of TF complexes that promote the opaque state (8, 46, 59). The addition of Ssn6 to Flo8 condensates led to a decrease in the size of these condensates even at Ssn6:Flo8 ratios as low as 1:100, and increasing the Ssn6 concentration further reduced Flo8 condensate size in a dose-dependent manner. Removal of the TPR or CTD did not block Ssn6 association with Flo8 condensates but did impact condensate properties. Together, these results suggest a model in which Ssn6 (± Tup1) can impact condensate behavior even at low Ssn6:TF ratios and that this modulatory property is mediated by both the TPR and CTD domains (Fig. 6).

### Ssn6 phase separation and gene expression

Condensate formation has been linked to gene activation at super-enhancers, where high concentrations of TFs, co-factors, and RNA Pol II co-localize (40, 60–63). In line with this, peptides that disrupt condensates or alter their dynamics can impair RNA Pol II recruitment and gene activation (64, 65). Conversely, condensates can actually hinder transcription at some promoters (66) and are implicated in gene silencing in compartments such as constitutive and facultative heterochromatin (67). It therefore appears that condensates play highly context-dependent roles in promoting or inhibiting gene expression at different loci.

Here, we show that Ssn6 reduces the size and liquid-like properties of a TF condensate in a dose-dependent manner. Given that condensation of white-opaque TFs has been linked to gene activation (7), the impact of Ssn6 on these condensates is expected to inhibit gene expression. This model adds to the existing repertoire of mechanisms by which Ssn6 can repress transcription as Ssn6−Tup1 can recruit histone deacetylases (HDACs), alter nucleosome positioning, and disrupt the transcriptional machinery (16, 20–22, 24–26). These mechanisms may be interconnected as evidence suggests that condensates can help recruit chromatin-modifying enzymes (68). It is also notable that a positive relationship exists between the number of DNA-binding TFs at a given locus and the level of Ssn6−Tup1 enrichment (69). High TF levels are expected to promote condensate formation and could, in turn, drive increased Ssn6−Tup1 recruitment to condensates at these loci. Finally, we emphasize that Ssn6 can disrupt TF condensate formation even in the absence of Tup1, a result that is consistent with Tup1-independent functions for *C. albicans* Ssn6 (11).

### A conserved role for condensate-modifying activities

Two mammalian homologs of Ssn6, UTX and UTY, promote embryonic development and tumor suppression and were recently shown to form condensates (68, 70–72). Phase separation of UTX was dependent on both a TPR and a central IDR, and these domains were necessary for transcription and tumor-suppressive activity (68). Interestingly, substitutions to the UTX IDR could abolish tumor-suppressive ability due to either (i) disrupting phase separation or (ii) increasing condensate size but decreasing their dynamic properties (68). This illustrates the complexity with which phase separation can impact gene expression and parallels the results shown here, where incorporation of Ssn6 into TF condensates reduced the size and/or liquid-like properties of condensates, with the expectation that this will inhibit TF activity. Taken together, these results suggest that the disordered domains of global repressors like Ssn6 and UTX/UTY can modulate the viscoelastic properties of transcriptional condensates as a means to control target gene expression.

### Conclusion

In this work, we show that both the TPR and CTD domains of Ssn6 play critical roles in regulating white-opaque switching, whereas the prion-like N and M domains play minimal roles in this process. While the TPR domain has long been recognized for mediating protein-protein interactions, we propose that the CTD modulates Ssn6 function via its effect on condensate formation (Fig. 6). This model parallels findings in mammalian cells where the phase-separating IDR of UTX/UTY plays a key role in the control of gene expression. The combination of a protein-protein interaction domain (e.g., a TPR) and a phase-modulating domain (e.g., a condensate-forming IDR) may represent a general mechanism for tuning transcription in the cell. In the case of Ssn6, further understanding how its individual domains modulate TF condensates will be critical for deciphering its role in regulating gene expression and fungal cell fate.

## MATERIALS AND METHODS

### Plasmid construction

Plasmids are described in Table S1 and oligonucleotides in Table S2. Plasmid sequences were confirmed by sequencing with Plasmidsaurus (https://plasmidsaurus.com). *C. albicans SSN6* sequences were PCR-amplified from SC5314 genomic DNA. Inserts for Ssn6∆N, Ssn6∆M, and Ssn6∆NM were assembled using fusion PCR. Here, two non-contiguous sequences were PCR-amplified with an overlap region added to one product, and these products fused together by PCR using the outermost primers. The resulting PCR products were inserted into pSFS2a (36) using the appropriate restriction enzymes. Ssn6∆T and Ssn6∆C inserts were incorporated by Golden Gate Assembly (GGA) (73) into a modified pSFS2a containing a BsaI landing pad (pSFS2a-BsaI). pSFS2a-BsaI was generated by annealing oligonucleotides 6048 and 6049 ligating them into pSFS2a digested with ApaI/XhoI.

For *E. coli* and mammalian cell expression, synthetic codon-optimized sequences for Ssn6, Tup1, and Flo8 were obtained from Biobasic (https://www.biobasic.com/) or Gene Universal (https://www.geneuniversal.com/). Sequences were inserted initially into an MBP vector (RB523, pRP1B–MBP/THMT). Details are provided in Tables S1 and S2. For mammalian cell expression, inserts were PCR-amplified and cloned into the eYFP-LacI vector (38).

To create CTD mutants, synthetic sequences were obtained from Gene Universal. Sequences were native or codon-optimized sequences depending on the desired application and were fused to the Ssn6∆C sequence using GGA. *C. albicans* sequences were cloned directly into pSFS2a-BsaI, while for the eYFP-LacI vector, sequences were assembled in pGGAselect and subsequently subcloned into eYFP-LacI.

### Strain construction

Details are included in Table S3. Oligonucleotides are listed in Table S2. Wild-type strains used were CAY6342 (white) and CAY6343 (opaque), which are equivalent to AHY135 and AHY136 that have been previously described (74). The *ssn6*/*SSN6* strain was CAY8083/AHY337 (6), and the *ssn6Δ/Δ* strain was TF121 (6). These strains were gifted by Dr. Alexander Johnson (University of California San Francisco). pSFS2a-*SSN6* plasmids were digested with restriction enzymes as described in Table S3 and then transformed into TF121. Correct integration was checked by PCR. Culture on Synthetic Complete Maltose medium was used to remove the nourseothricin-resistance marker (36), after which the strains were tested for nourseothricin susceptibility. Ssn6-mNeonGreen strains were constructed by PCR-amplifying an mNeonGreen cassette from RB895 using primers containing 75 bp homology to the destination site and 25 bp homology to the mNeonGreen cassette and transformed into target strains. The putative phosphorylation sites mutated in the Ssn6 CTD were S885, S887, T889, S894, S1027, and S1028.

### White-opaque switching assays

Cells of a single phenotypic state were taken from yeast extract peptone dextrose (YPD) plates and resuspended in phosphate-buffered saline (PBS). Cells per milliliter were estimated using optical density with 1 OD_600_ = 2 × 10^7^ cells/mL. Suspensions were serially diluted in PBS to 2 × 10^3^ cells/mL. Approximately 100 cells were plated in triplicate on synthetic complete dextrose (SCD) medium. Plates were scored after incubation at 22°C for 7 days.

### *C. albicans* cell imaging

For fluorescence and differential interference contrast microscopy (DIC) imaging, cells were taken from plates and suspended in PBS and then imaged using a Zeiss Axio Observer Z1 microscope equipped with Zen software (Zen v.3.0 blue edition). For Hoechst staining, cells were pre-incubated for 1 h with 1 mg/mL of Hoechst 33258, then pelleted and resuspended in fresh PBS before imaging.

### Mammalian cell culture, live-cell imaging, and LacO array analysis

U2OS cells containing a LacO array (~50,000 LacO elements) were obtained from the Tjian Lab (38). U2OS cells were cultured, transfected, and imaged as described (7). LacI focus area size and intensity were measured by masking the YFP signal and analyzing particles in FIJI (75). Background fluorescence intensity was corrected by subtracting YFP signal outside the array spot in the cell nucleus. To quantify mCherry–PrLD enrichment at the LacO array bound by eYFP-LacI constructs, we followed a method similar to that of Chong et al. (38). The array spot was masked in the YFP channel, and the signal was measured in the mCherry channel. Two locations near the array in the mCherry channel were then measured and averaged to represent average background signal in the nucleus. The mCherry enrichment was then calculated by dividing the array mCherry signal by the background signal. Hexanediol treatment was conducted by adding 20% 1,6-hexanediol dissolved in pre-warmed Dulbecco’s modified Eagle medium to each well in a 1:1 ratio, for a final concentration of 10% 1,6-hexanediol. Images were taken for 5 min with intervals of 10 s. Quantification was conducted by masking the YFP channel in FIJI and then analyzing the number of particles in each frame.

### Protein purification

Proteins were purified as described previously (7). His–MBP fusion protein constructs were transformed into BL21 (DE3) Star *E. coli* cells for expression. Cells were grown at 37°C overnight, then diluted 1:100 into fresh Luria broth medium, cultured at 37°C until they reached an OD_600_ of 0.5–0.7, and then induced with 1 mM isopropyl β-d-1-thiogalactopyranoside. Ssn6 protein variants were induced at 16°C overnight. Induction conditions for other MBP-fusion proteins were 30°C for 4 h. Cells were lysed with lysozyme followed by sonication in lysis buffer consisting of 10 mM Tris pH 7.4, 1 M NaCl, 1 mM phenylmethylsulfonyl fluoride, and a protease inhibitor cocktail (Thermo Scientific Pierce Protease Inhibitor). Proteins were purified by nickel affinity chromatography, followed by size exclusion using a Sephacryl S300 26/60 column (GE Healthcare). Fractions were concentrated using Amicon Ultra 50K concentrators (Millipore) and snap-frozen in liquid nitrogen.

### Phase separation assays

Protein stocks were thawed at 22°C and diluted in 10 mM Tris-HCl, pH 7.4, and 150 mM NaCl, and then concentrated in centrifugal filter units (Amicon Ultra—0.5 mL centrifugal filter units) to 100 µL. Protein concentration was measured using a Nanodrop 2000c (Thermo Fisher Scientific). Proteins were then diluted in 10 mM Tris-HCl buffer with 150 mM NaCl to the desired concentrations. Protein reactions with TEV protease were set up in 10 µL total volumes (9.5 µL protein with 0.5 µL of 0.3 mg/mL TEV) and incubated for 30 min at room temperature (76). Proteins were cleaved with TEV prior to addition of any PEG. Labeling of proteins with DyLight or AlexaFluor fluorescent dyes (Thermo Fisher Scientific) was carried out according to the manufacturer’s instructions. Labeled proteins were added to assays at concentrations below 0.5 µM before TEV incubation. Proteins were imaged immediately after incubation on chamber slides (SPI Supplies, 10-chamber slides) with 0.8 µL solution per chamber and sealed with a glass coverslip. All images were acquired at 63× magnification with a 1.6× Optovar using a Zeiss Axio Observer Z1 inverted fluorescence microscope. Post-imaging processing was carried out in FIJI (ImageJ). Quantification of condensate size and shape properties was conducted by masking the fluorescent channel in FIJI, analyzing particles, and then measuring the relevant parameters.

### RNA sequencing

*C. albicans* cells taken from YPD plates were inoculated into 15 mL SCD and incubated for 18 h in a rotator at 22°C. Cells per milliliter were determined using optical density with 1 OD_600_ = 2 × 10^7^ cells/mL, and a volume equivalent to 10 OD_600_ of cells was harvested by pelleting, then snap-frozen with liquid nitrogen, and stored at −80°C. RNA extraction was conducted using the RiboPure Yeast Kit (Invitrogen). Library preparation was conducted using the QuantSeq 3′ mRNA-Seq Library Prep Kit FWD for Illumina (Lexogen), with unique i7 barcodes applied for each sample. Library concentrations were measured using a Qubit Fluorometer (Invitrogen) and then multiplexed. The final pooled library was sequenced on an Illumina NextSeq as SR85 and de-multiplexed by the UC Davis Genome Center.

### Analysis of RNA sequencing

Fasta reads were trimmed with BBDuk and aligned using STAR aligner (77). Assembly 21 chromosome FASTA files and GFF index file were downloaded from the Candida Genome Database (78). An edited GFF file to extend the 3′ regions of genes was kindly provided by Deepika Gunasekaran (UCMerced). Reads were normalized in R version 4.3.1 using the edgeR package (79), and differential gene expression analysis was performed. Principal component analysis (PCA) was performed in R using factoextra (80) and visualized using ggplot2 (81). Heatmap visualization was performed in R using pheatmap (82).

## Data Availability

Sequencing data were submitted to the NCBI Gene Expression Omnibus (GEO; https://www.ncbi.nlm.nih.gov/geo/) database under accession number GSE319560.

## SUPPLEMENTAL MATERIAL

Supplemental Material mbio.00316-26-s0001.pdf Fig. S1 to S3 and Tables S1 to S3.

ASM does not own the copyrights to Supplemental Material that may be linked to, or accessed through, an article. The authors have granted ASM a non-exclusive, world-wide license to publish the Supplemental Material files. Please contact the corresponding author directly for reuse.
