# Supplementary material for: The transcriptional repressor Ssn6 modulates phase separation to regulate fungal gene expression
Source: mBio. 2026 Mar 30;17(5):e00316-26. doi: 10.1128/mbio.00316-26 (PMC13170171; doi:10.1128/mbio.00316-26)
Supplement: Supplemental Material — Fig. S1 to S3 and Tables S1 to S3. [file mbio.00316-26-s0001.pdf]

Supplementary Data

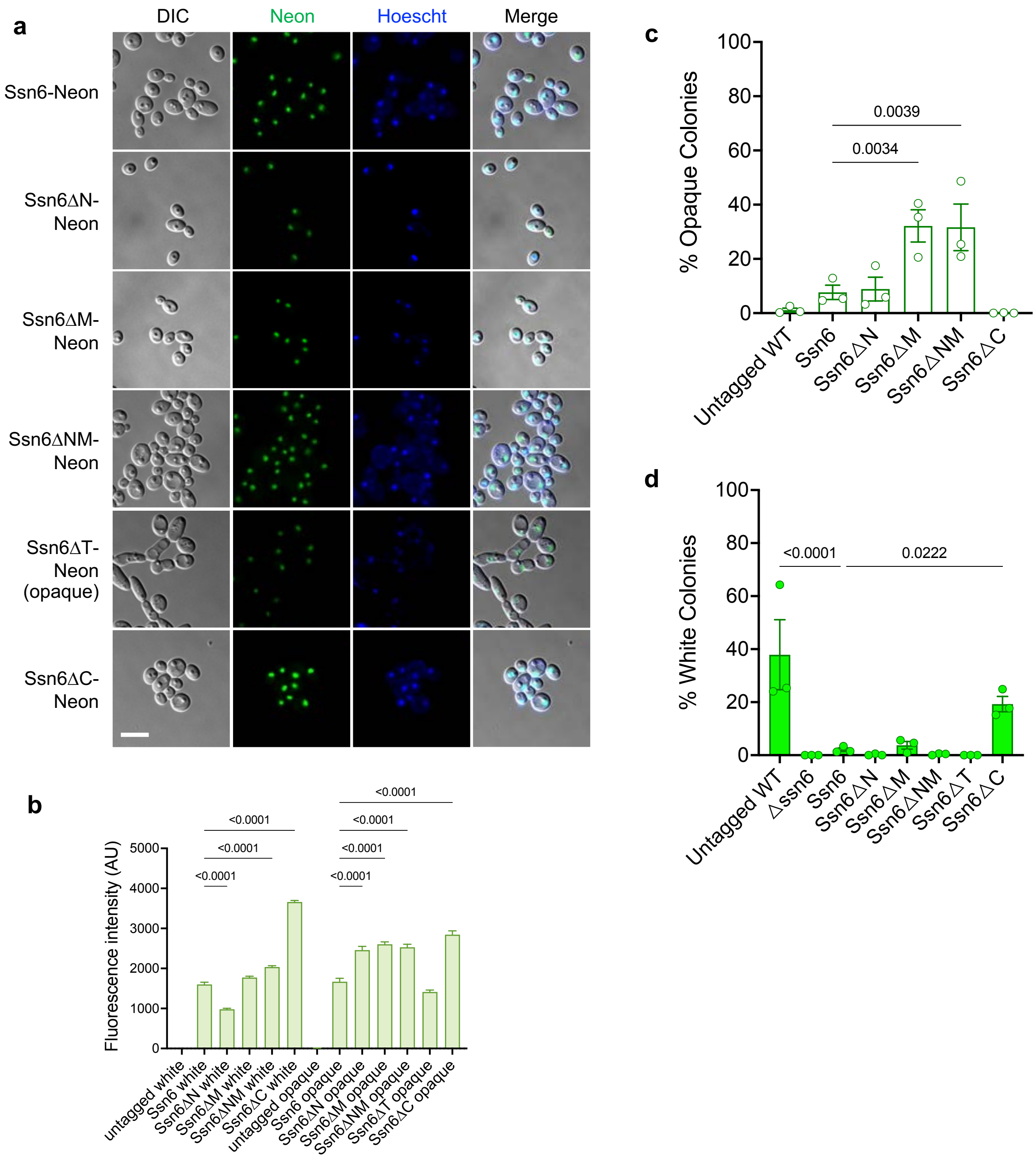

Supplementary Figure 1: Quantification of Ssn6 expression in *C. albicans* cells. a. Ssn6-Neon strains stained with Hoescht. Images shown are for white cells unless marked. Scale bar, 10 μm. **b.** Quantification of data in panel A. **c.** White-to-opaque switching assays in strains expressing Ssn6 variants. **d.** Opaque-to-white switching assays in strains expressing Ssn6 variants. Statistics shown are those significant versus the full-length Ssn6-Neon strain in the respective cell type, Fisher's LSD test, $p \leq 0.05$.

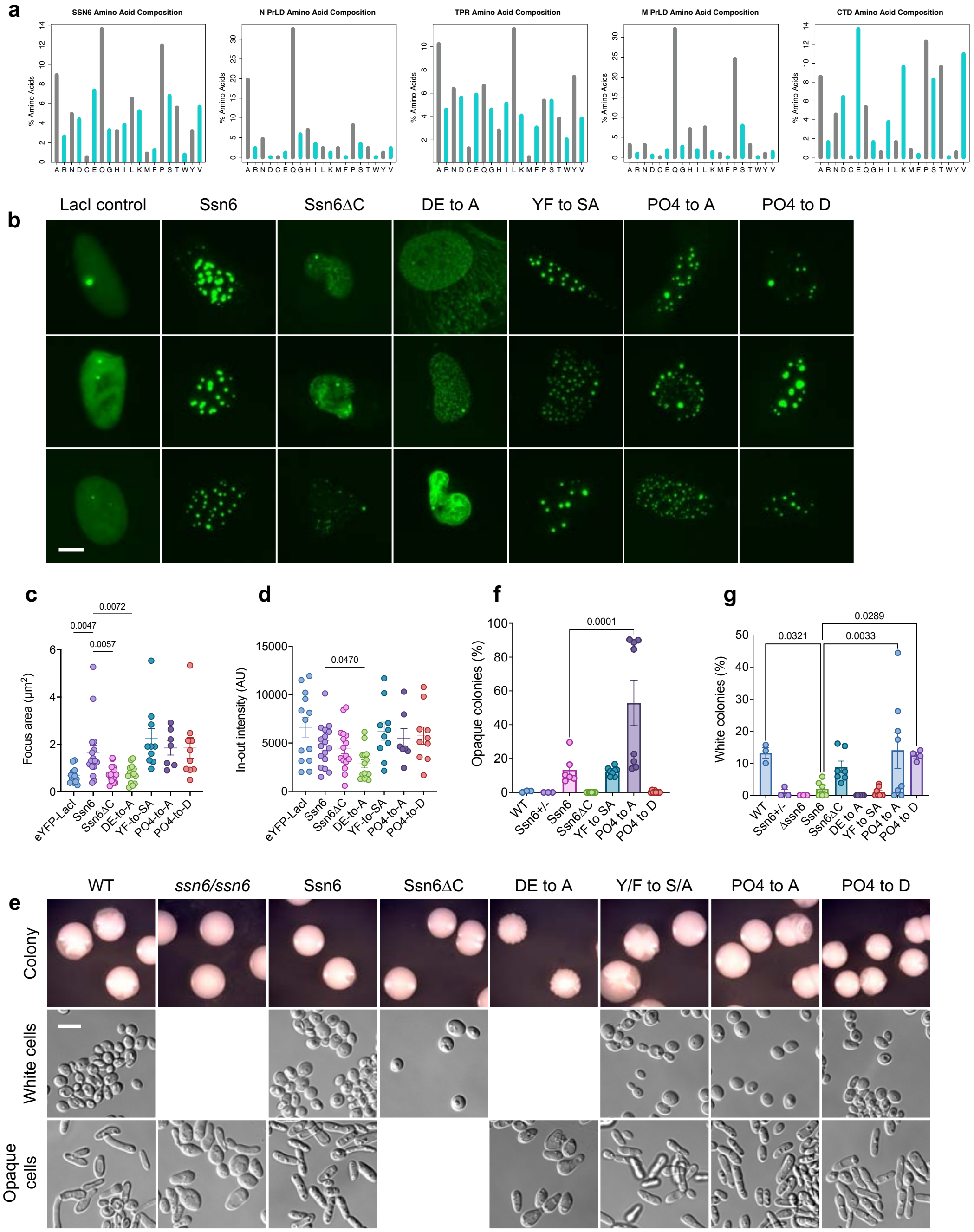

Supplementary Figure 2: Analysis of Ssn6 CTD variants for condensate formation in U2OS cells and for white-opaque switching properties in *C. albicans* cells. a. Amino acid composition of each Ssn6 domain. **b.** Expression of Ssn6 CTD variants (fused to eYFP-LacI) in U2OS cells. Mutations to specific amino acid types were made only to residues in the CTD. DE to A; acidic residues (aspartate/glutamate) substituted with alanine. YF to SA; aromatic residues (tyrosine/phenylalanine) substituted with serine/alanine. PO4 to A; potential phosphorylation sites substituted with alanine. PO4 to D; potential phosphorylation sites substituted with aspartate. **c.** Size quantification of puncta from panel b. **d.** Intensity quantification of puncta from panel b. **e.** Colony (top) and cell (middle and bottom) images of Ssn6 CTD mutants. **f.** White-to-opaque switching data for Ssn6 CTD mutants. **g.** Opaque-to-white switching data for Ssn6 CTD mutants. Statistics are shown for Fisher's LSD test against the Ssn6 strain, p ≤ 0.05. Scale bars, 10μm.

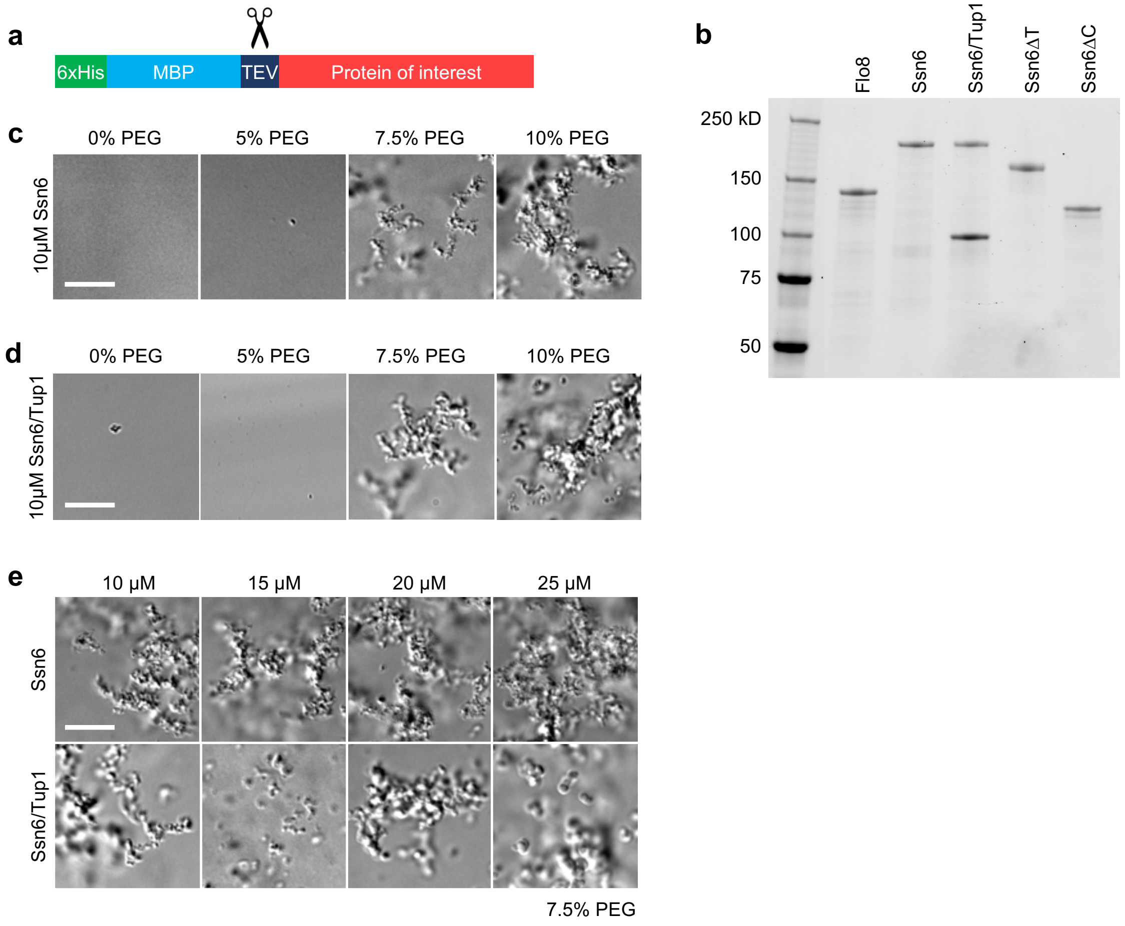

Supplementary Figure 3: Ssn6 forms protein aggregates *in vitro* in the presence of PEG, both with and without Tup1. a. Schematic of MBP fusion proteins purified from *E. coli*. **b.** SDS-PAGE of MBP fusion proteins purified for Fig. 4 and Supplementary Fig. 3. In the Ssn6/Tup1 lane the upper band is MBP-Ssn6 and the lower band if MBP-Tup1. **c.** MBP-Ssn6 (10 μM) with various PEG8000 concentrations. **d.** Co-purified MBP-Ssn6/MBP-Tup1 (10 μM) incubated with various PEG8000 concentrations. **e.** Different concentrations of MBP-Ssn6 alone or co-purified with MBP-Tup1 at 7.5% PEG8000. Scale bars, 10 μm.

Tables

Supplementary Table 1. Plasmids used in this study.

Plasmid	Name	Method	Starting Vector	Inserts	Template	Oligos	Restriction enzymes
pSFS2a	pSFS2a	Reuss et al. 2002					
RB523	MBP vector, pRP1B–MBP/THMT	Frazer et al., 2020, gift from Nick Fawzi					
RB1207	mCherry vector	Chong et al., 2018, gift from Tjian lab					
RB1208	eYFP-LacI vector	Chong et al., 2018, gift from Tjian lab					
RB1397	pSFS2a-BsaI	Oligo insertion	pSFS2a	Oligos 6048/6049			ApaI, XhoI
RB895	pSFS2a-mNeon vector	Subcloning	pSFS2a	mNeon	RB880 (synthetic)	n/a	insert: XhoI; vector: XhoI, CIP
RB1277	pSFS2a-Ssn6	Cloning	pSFS2a	5'-Ssn6-3'	SC5314 gDNA	5607, 5608	ApaI, XhoI
		Cloning		far 3'	SC5314 gDNA	5741, 5742	SacI-HF, SacII
RB1279	pSFS2a-Ssn6ΔN	Fusion PCR	pSFS2a	5'	SC5314 gDNA	5607, 5609	
				Ssn6ΔN-3'	SC5314 gDNA	5610, 5608	
				5'-Ssn6ΔN-3'	5' and Ssn6ΔN-3'	5607, 5608	ApaI, XhoI
		Cloning		far 3'	SC5314 gDNA	5741, 5742	SacI-HF, SacII
RB1281	pSFS2a-Ssn6ΔM	Fusion PCR	pSFS2a	5'-Ssn6NT	SC5314 gDNA	5607, 5611	
				Ssn6C-3'	SC5314 gDNA	5612, 5608	
				5'-Ssn6ΔM-3'	5'-Ssn6NT and Ssn6C-3'	5607, 5608	ApaI, XhoI
		Cloning		far 3'	SC5314 gDNA	5741, 5742	SacI-HF, SacII
RB1283	pSFS2a-Ssn6ΔNM	Fusion PCR	pSFS2a	5'	SC5314 gDNA	5607, 5609	
				Ssn6T	SC5314 gDNA	5610, 5611	
				Ssn6C-3'	SC5314 gDNA	5612, 5608	
				5'-Ssn6ΔNM-3'	5' and Ssn6T and Ssn6C-3'	5607, 5608	ApaI, XhoI
		Cloning		far 3'	SC5314 gDNA	5741, 5742	SacI-HF, SacII
RB1958	pSFS2a-Ssn6ΔT	Golden Gate	pSFS2a-BsaI	5'-Ssn6N	RB1277	6905, 6906	BsaI-HF-v2
				Ssn6MC-3'	RB1277	6907, 6910	BsaI-HF-v2

		Cloning		far 3'	SC5314 gDNA	5741, 5742	SacI-HF, SacII
RB1960	pSFS2a-Ssn6∆C	Golden Gate	pSFS2a-BsaI	5'-Ssn6∆C	RB1277	6905, 6908	BsaI-HF-v2
				3'	RB1277	6909, 6910	BsaI-HF-v2
		Cloning		far 3'	SC5314 gDNA	5741, 5742	SacI-HF, SacII
RB1249		Cloning	RB523	Ssn6∆N	RB1170	5613, 5614	NdeI, XhoI
RB1251*		Cloning	RB1170	Ssn6∆M	RB1254	n/a	BamHI, XhoI
RB1254		Fusion PCR	RB523	Ssn6T	RB1170	5613, 5616	
				Ssn6C	RB1170	5617, 5614	
				Ssn6∆NM	Ssn6T and Ssn6C	5613, 5614	NdeI, XhoI
RB1462	eYFP-Ssn6-LacI	Cloning	RB1208	Ssn6	RB1170	6038, 6107	insert: BsrGI-HF & XmaI; vector: BsrGI-HF & BspEI
RB1464	eYFP-Ssn6∆N-LacI	Cloning	RB1208	Ssn6∆N	RB1249	6039, 6107	insert: BsrGI-HF & XmaI; vector: BsrGI-HF & BspEI
RB1466	eYFP-Ssn6∆M-LacI	Cloning	RB1208	Ssn6∆M	RB1251	6038, 6107	insert: BsrGI-HF & XmaI; vector: BsrGI-HF & BspEI
RB1468	eYFP-Ssn6∆NM-LacI	Cloning	RB1208	Ssn6∆NM	RB1253	6039, 6107	insert: BsrGI-HF & XmaI; vector: BsrGI-HF & BspEI
RB1956	eYFP-Ssn6∆T-LacI	Cloning	RB1208	Ssn6∆T	RB1734 (Twist Biosciences)	6038, 6107	insert: BsrGI-HF & XmaI; vector: BsrGI-HF & BspEI
RB1957	eYFP-Ssn6∆C-LacI	Cloning	RB1208	Ssn6∆C	RB1170	6038, 6106	BsrGI-HF, BspEI
RB2024	eYFP-Ssn6-N-LacI	Cloning	RB1208	Ssn6N	RB1170	6038, 6153	insert: BsrGI-HF & XmaI; vector: BsrGI-HF & BspEI
RB2025	eYFP-Ssn6-T-LacI	Cloning	RB1208	Ssn6T	RB1170	6039, 6154	insert: BsrGI-HF & XmaI; vector: BsrGI-HF & BspEI
RB2026	eYFP-Ssn6-M-LacI	Cloning	RB1208	Ssn6M	RB1170	6155, 6156	insert: BsrGI-HF & XmaI; vector: BsrGI-HF & BspEI
RB2027	eYFP-Ssn6-C-LacI	Cloning	RB1208	Ssn6C	RB1170	6157, 6107	insert: BsrGI-HF & XmaI; vector: BsrGI-HF & BspEI
RB2028	eYFP-Ssn6-NT-LacI	Cloning	RB1208	Ssn6NT	RB1170	6038, 6154	insert: BsrGI-HF & XmaI; vector: BsrGI-HF & BspEI
RB2029	eYFP-Ssn6MC-LacI	Cloning	RB1208	Ssn6MC	RB1170	6155, 6107	insert: BsrGI-HF & XmaI; vector: BsrGI-HF & BspEI
RB2182	eYFP-Ssn6-CTD-DE-to-A-LacI	Golden Gate, then subcloning	pGGAselect, then RB1208	Ssn6∆C	RB1462	8579, 8580	BsmBI-HFv2, then BsrGI-HF & BspEI

				CTD-DE-to-A	RB2168 (Gene Universal)	8581, 8582	BsmBI-HFv2, then BsrGI-HF & BspEI
RB2183	eYFP-Ssn6-CTD-YF-to-SA-LacI	Golden Gate, then subcloning	pGGAselect, then RB1208	Ssn6ΔC	RB1462	8579, 8583	BsmBI-HFv2, then BsrGI-HF & BspEI
				CTD-YF-to-SA	RB2169 (Gene Universal)	8584, 8585	BsmBI-HFv2, then BsrGI-HF & BspEI
RB2184	eYFP-Ssn6-CTD-PO4-to-Ala-LacI	Golden Gate, then subcloning	pGGAselect, then RB1208	Ssn6ΔC	RB1462	8579, 8586	BsmBI-HFv2, then BsrGI-HF & BspEI
				CTD-PO4-to-A	RB2170 (Gene Universal)	8587, 8588	BsmBI-HFv2, then BsrGI-HF & BspEI
RB2185	eYFP-Ssn6-CTD-PO4-to-Asp-LacI	Golden Gate, then subcloning	pGGAselect, then RB1208	Ssn6ΔC	RB1462	8579, 8586	BsmBI-HFv2, then BsrGI-HF & BspEI
				CTD-PO4-to-D	RB2171 (Gene Universal)	8587, 8588	BsmBI-HFv2, then BsrGI-HF & BspEI
RB2178	pSFS2a-Ssn6-CTD-DE-to-A	Golden Gate	pSFS2a-BsaI	5'-Ssn6ΔC	RB1277	8567, 8568	BsaI-HF-v2
				CTD-DE-to-A	RB2164 (Gene Universal)	8569, 8570	BsaI-HF-v2
				3'	RB1277	8571, 8572	BsaI-HF-v2
		Cloning		far 3'	SC5314 gDNA	5741, 5742	SacI-HF, SacII
RB2179	pSFS2a-Ssn6-CTD-YF-to-SA	Golden Gate	pSFS2a-BsaI	5'-Ssn6ΔC	RB1277	8567, 8573	BsaI-HF-v2
				CTD-YF-to-SA	RB2165 (Gene Universal)	8574, 8575	BsaI-HF-v2
				3'	RB1277	8576, 8572	BsaI-HF-v2
		Cloning		far 3'	SC5314 gDNA	5741, 5742	SacI-HF, SacII
RB2180	pSFS2a-Ssn6-CTD-PO4-to-Ala	Golden Gate	pSFS2a-BsaI	5'-Ssn6ΔC	RB1277	8567, 8573	BsaI-HF-v2
				CTD-PO4-to-A	RB2166 (Gene Universal)	8574, 8577	BsaI-HF-v2
				3'	RB1277	8578, 8572	BsaI-HF-v2
		Cloning		far 3'	SC5314 gDNA	5741, 5742	SacI-HF, SacII
RB2181	pSFS2a-Ssn6-CTD-PO4-to-Asp	Golden Gate	pSFS2a-BsaI	5'-Ssn6ΔC	RB1277	8567, 8573	BsaI-HF-v2

				CTD-PO4-to-D	RB2167 (Gene Universal)	8574, 8577	BsaI-HF-v2
				3'	RB1277	8578, 8572	BsaI-HF-v2
		Cloning		far 3'	SC5314 gDNA	5741, 5742	SacI-HF, SacII
RB1619	mCherry-Tup1	Cloning	pmCherry (RB1207)	Tup1	RB1172	6282, 6283	insert: BamHI-HF & XmaI; vector: BamHI-HF & BspEI
RB1170	MBP-Ssn6	Subcloning	RB523	Ssn6	synthetic	n/a	NdeI & XhoI
RB1172	MBP-Tup1	Subcloning	RB523	Tup1	synthetic	n/a	NdeI & XhoI
RB1227	MBP-Tup1-CmR	Subcloning	RB862 (MBP chloramphenicol vector)	Tup1	RB1172	n/a	NdeI & XhoI
RB514	MBP-Efg1	Cloning	RB523	Efg1	synthetic	n/a	NdeI & XhoI
RB971	MBP-Flo8	Subcloning	RB523	Flo8	RB959 (synthesized)	n/a	NdeI & XhoI
RB2258	MBP-Ssn6ΔT	Golden Gate, then subcloning	pGGAselect, then RB523	Ssn6N	RB1171	8782, 8783	BsmBI-HFv2, then NdeI & XhoI
				Ssn6MC	RB1171	8784, 8785	BsmBI-HFv2, then NdeI & XhoI
RB2257	MBP-Ssn6ΔC	Cloning	RB523	Ssn6ΔC	RB1171	5615, 8781	NdeI & XhoI

*RB1251 construction used an existing BamHI site in TPR region to add portion of Ssn6ΔNM into full Ssn6 sequence to create Ssn6ΔM.

Supplementary Table 2. Oligonucleotides used in this study.

Oligo #	Oligo name	Oligo sequence
4439	pSFS2 Right	GCGAAAAAGTGGGCACTAAG
5091	Neon rev	TTTATACAATTCATCCATACCCATAACATCA
5607	Ssn6 5 fwd ApaI	GGACCGGGGCCCCAGAAAGACAAAGAGAGAGCG
5608	Ssn6 3 rev XhoI	GGACCGCTCGAGCAACTATATTCCATCCAGAAAATATTTCTATC
5609	Ssn6 5 Rev	CATCTTTCCGGATCTTTGTTAATGA
5610	Ssn6 delta NPrLD Fwd	TCATTAACAAAGATCCGGAAAGATGTTACCTAGTTCAGCTGCTCT TAAT
5611	Ssn6 5 and N-ORF Rev	ATACTTTGTCAATTGTTCTAATCTTGC
5612	Ssn6 C-ORF Fwd overlaps N	GCAAGATTAGAACAATTGACAAAGTATATTGCACCTGTTAGACAA GAAC
5613	MBP-Ssn6 dN-PrLD Fwd NdeI	GGACCGCATATGCTGCCGTCTAGCGCCG
5614	MBP-Ssn6 dN-PrLD Rev XhoI	GGACCGCTCGAGTTATTCGTCATCATAGTTTTCGTCTTC
5615	MBP-Ssn6 Fwd NdeI	GGACCGCATATGTATGCTACCGCGCATACC
5616	MBP-Ssn6 N-ORF rev	GTATTTGGTCAGCTGTTCAAGAC
5617	MBP-Ssn6 C-ORF Fwd	GTCTTGAACAGCTGACCAAATACATTGCACCAGTGCGTCAG
5683	Ssn6-AB check left fwd	TCTTCTCTGCCCCATAAAATTCA
5684	Ssn6-AB check right rev	CACTAATACCATTACCAACAGTTTTCAA
5740	Ssn6 tail fwd (SacII)	GGACCGCCGCGGATTTGACTATCTTGTGAATTAAAGTTTCAA
5741	Ssn6 tail rev (SacI)	GGACCGGAGCTCTATTGCATGTTTTAAAAATGATTATTCACAAAC
5742	CaWor1 FL rev no stop XmaI	GGACCGCCCGGGAGTACCGGTGTAATACGACC
5799	Ssn6 tail check rev	TTTCATTGGGAAAGAACAAGACAATAAAG
6038	EYFP Ssn6 fwd	ggaccgtgtacaagggtggtagtggttatgctaccgcgcataccattaaac
6039	EYFP Ssn6 deltaN fwd	ggaccgtgtacaagggtggtagtggtctgccgtctagcgccgcactg
6048	GGA Adapter for pSFS2A Fwd	CCAGTTGAGACCCATTAATGGTCTCTTACTC
6049	GGA Adapter for pSFS2A Rev	TCGAGAGTAAGAGACCATTAATGGGTCTCAACTGGGGCC
6106	mCherry ssn6 rev 2	ggaccgctcgagctatacctttctcttcttttttggatcttcgtcatcatagttttcgtc
6107	LacI Ssn6 rev 2	ggaccgcccgggttcgtcatcatagttttcgtcttcttc
6153	Ssn6 N PrLD LacI rev	GGACCGTCCGGAACGCTGTTGTTGCTGCTG
6154	Ssn6 TPR LacI rev	GGACCGTCCGGAGTATTTGGTCAGCTGTTCAAG
6155	Ssn6 M PrLD eYFP fwd	GGACCGTGTACAAGGGTGGTAGTGGTCAGCAGGAAGGCAACAC CC
6156	Ssn6 M PrLD LacI rev	GGACCGTCCGGAGTGTGGCTGCTGGTACTGCTG
6283	Tup1 BFP mCherry rev	ggaccgggatccctatacctttctcttcttttttggatctttttggtccatttccag
6342	Ssn6 N PrLD rev	tcgctgttgttgctgctgttgttgttgttggg
6350	Ssn6 upstream-C fusion fwd	caaagatccggaaagatgattgcacctgttagac
6455	Ssn6 addback T mid seq fwd	gtgaagtatggtatgatttgggg
6905	GG Ca Ssn6-5N fwd	GGCTACGGTCTCTCAGTCAGAAAGACAAAG AGAGAGCG
6906	GG Ca Ssn6-5N rev	GGCTACGGTCTCTCCTTCTTGTTGTCGCTGTTGTTGCTGCTG
6907	GG Ca Ssn6-MC3_fwd	GGCTACGGTCTCTAAGGTAATACTCACCCA C
6908	GG Ca Ssn6-deltaC_rev	GGCTACGGTCTCATCTCTAATGTGGTTGTT GATATTG
6909	GG Ca Ssn6-3near_fwd	GGCTACGGTCTCAGAGATAAAGAAAAGTGG TGAAATTG
6910	GG Ca Ssn6-3near_rev	GGCTACGGTCTCCAGTACAACTATATTCCA TCCAGAAAATATTTC
7113	Ssn6 neon tag long oligo fwd	gatgacgtcaaaaaagatgaaaatccagaacctccaatgagaaagattgaagaagatgaa aattatgatgatgaaGGTGGTAGTGGTATGGTTTCTAAAG
7114	Ssn6 delta C neon tag long oligo fwd	ttacacaactctgctaatatcatatcagctccatcacaagtacctcaaccacaacaacaatatc aacaaccacatGGTGGTAGTGGTATGGTTTCTAAAG
7115	Ssn6 neon tag rev long oligo	ccccatccccctaccccgtcaaatatatatatatattcaactatcatatacaatttcaccacttttcttt atctggcggccgctctagaactagtggatc

8036	Ca Ssn6 N check rev	cttgcatgtagtccaccg
8567	GG Ca Ssn6 5 fwd	ggctacggtctcgcagtcagaaagacaaagagagag
8568	GG Ca Ssn6 M PrLD DE to A rev	ggctacggtctcgtctaacaggtgcaatatgtggttgttgatattgttg
8569	GG Ca Ssn6 CTD DE to A fwd	ggctacggtctcgtagacaagctcaagttaac
8570	GG Ca Ssn6 CTD DE to A rev	ggctacggtctccctttatctctaagcagcagcataattag
8571	GG Ca Ssn6 3 DE to A fwd	ggctacggtctccaaagaaaagtggtgaaattg
8572	GG Ca Ssn6 3 rev	ggctacggtctccagtacaactatattccatccagaaaatatttc
8573	GG Ca Ssn6 M PrLD rev	ggctacggtctcatgatattgttgttgtggttg
8574	GG Ca Ssn6 CTD fwd	ggctacggtctcaatcaacaaccacatattgcacctgttagacaag
8575	GG Ca Ssn6 CTD YF to SA rev	ggctacggtctcctctattcatcatctgaattttcatc
8576	GG Ca Ssn6 3 YF to SA fwd	ggctacggtctcctagagataaagaaaagtggtgaaattg
8577	GG Ca Ssn6 CTD PO4 rev	ggctacggtctcgtctattcatcatcataattttcatcttc
8578	GG Ca Ssn6 3 PO4 fwd	ggctacggtctcgtagagataaagaaaagtggtgaaattg
8579	GG Ec Ssn6 BsrGI fwd	ggctaccgtctccggagtgtacaagggtggtagtgg
8580	GG Ec Ssn6 M DE to A rev	ggctaccgtctcctggctgctggtactgctg
8581	GG Ec Ssn6 CTD DE to A fwd	ggctaccgtctccgccacacattgcaccagtgcgtcaggc
8582	GG Ec Ssn6 CTD DE to A rev	ggctaccgtctctatggtccggaggcggcggcata
8583	GG Ec Ssn6 M YF to SA rev	ggctaccgtctcatggctgctggtactgctg
8584	GG Ec Ssn6 CTD YF to SA fwd	ggctaccgtctcagccacacattgcaccagtgcgtcag
8585	GG Ec Ssn6 CTD YF to SA rev	ggctaccgtctctatggtccggattcgtcatcgctg
8586	GG Ec Ssn6 M PO4 rev	ggctaccgtctcgtggctgctggtactgctg
8587	GG Ec Ssn6 CTD PO4 fwd	ggctaccgtctcggccacacattgcaccagtgcgtcag
8588	GG Ec Ssn6 CTD PO4 rev	ggctaccgtctctatggtccggattcgtcatcatagttttc
8781	MBP-Ssn6deltaC Ec XhoI rev	ggaccgctcgagTTAGTGTGGCTGCTGGTACTGC
8782	GG MBP-Ssn6 Ec NdeI fwd	ggctaccgtctctggagcatatgtatgctaccgcgcATACC
8783	GG Ec Ssn6 N rev	ggctaccgtctcctgttgccttcctgctgacgctgttgttgctgctg
8784	GG Ec Ssn6 M fwd	ggctaccgtctccaacacccatccgccgcag
8785	GG MBP-Ssn6 Ec XhoI rev	ggctaccgtctcaatggctcgagttattcgtcatcatagttttcgtcttcTTC

Supplementary Table 3. Strains used in this study.

Strain #	name	description
CAY6342	WT white	AHY135 from Lohse, et al. 2013, *PNAS.*
CAY6343	WT opaque	AHY136 from Lohse, et al. 2013, *PNAS.*
TF121	*ssn6/ssn6*	TF121 from Lohse, et al. 2016, *Genetics.*
CAY8083	*ssn6*/SSN6	AHY337 from Hernday, et al. 2016, *mBio*.
CAY11369	Ssn6 full with 3' homology satS	RB1277 digested with ApaI and SacI and transformed into TF121. Integration checked with oligos 5683 & 5611. 5' integration checked with oligos 5740 & 5799. SAT resistance cassette flipped out with maltose media.
CAY11371	Ssn6 full with 3' homology satS	RB1277 digested with ApaI and SacI and transformed into TF121. Integration checked with oligos 5683 & 5611. 5' integration checked with oligos 5740 & 5799. SAT resistance cassette flipped out with maltose media.
CAY11375	Ssn6ΔN with 3' homology satS	RB1279 digested with ApaI and SacI and transformed into TF121. Integration checked with oligos 5683 & 5611. 5' integration checked with oligos 5740 & 5799. SAT resistance cassette flipped out with maltose media.
CAY11377	Ssn6ΔN with 3' homology satS	RB1279 digested with ApaI and SacI and transformed into TF121. Integration checked with oligos 5683 & 5611. 5' integration checked with oligos 5740 & 5799. SAT resistance cassette flipped out with maltose media.
CAY11381	Ssn6ΔM with 3' homology satS	RB1281 digested with ApaI and SacI and transformed into TF121. Integration checked with oligos 5683 & 5611. 5' integration checked with oligos 5740 & 5799. SAT resistance cassette flipped out with maltose media.
CAY11383	Ssn6ΔM with 3' homology satS	RB1281 digested with ApaI and SacI and transformed into TF121. Integration checked with oligos 5683 & 5611. 5' integration checked with oligos 5740 & 5799. SAT resistance cassette flipped out with maltose media.
CAY11387	Ssn6ΔNM with 3' homology satS	RB1283 digested with ApaI and SacI and transformed into TF121. Integration checked with oligos 5683 & 5611. 5' integration checked with oligos 5740 & 5799. SAT resistance cassette flipped out with maltose media.
CAY11389	Ssn6ΔNM with 3' homology satS	RB1283 digested with ApaI and SacI and transformed into TF121. Integration checked with oligos 5683 & 5611. 5' integration checked with oligos 5740 & 5799. SAT resistance cassette flipped out with maltose media.
CAY13592	Ssn6ΔT with 3' homology satS	RB1958 digested with ApaI and SacI and transformed into TF121. Integration checked with oligos 5683/6342 (5') and 4439/5799 (3'). SAT resistance cassette flipped out with maltose media.
CAY13593	Ssn6ΔT with 3' homology satS	RB1958 digested with ApaI and SacI and transformed into TF121. Integration checked with oligos 5683/6342 (5') and 4439/5799 (3'). SAT resistance cassette flipped out with maltose media.
CAY13594	Ssn6ΔC with 3' homology satS	RB1960 digested with ApaI and SacI and transformed into TF121. Integration checked with oligos 5683/6342 (5') and 4439/5799 (3'). SAT resistance cassette flipped out with maltose media.
CAY13595	Ssn6ΔC with 3' homology satS	RB1960 digested with ApaI and SacI and transformed into TF121. Integration checked with oligos 5683/6342 (5') and 4439/5799 (3'). SAT resistance cassette flipped out with maltose media.

CAY14850	Ssn6 CTD DE to A satS	RB2178 digested with PspOMI and SacI and transformed into TF121. Integration checked with oligos 5683/8036 (5') and 4439/5799 (3'). SAT resistance cassette flipped out with maltose media.
CAY14851	Ssn6 CTD DE to A satS	RB2178 digested with PspOMI and SacI and transformed into TF121. Integration checked with oligos 5683/8036 (5') and 4439/5799 (3'). SAT resistance cassette flipped out with maltose media.
CAY14852	Ssn6 CTD YF to SA satS	RB2179 digested with PspOMI and SacI and transformed into TF121. Integration checked with oligos 5683/8036 (5') and 4439/5799 (3'). SAT resistance cassette flipped out with maltose media.
CAY14853	Ssn6 CTD YF to SA satS	RB2179 digested with PspOMI and SacI and transformed into TF121. Integration checked with oligos 5683/8036 (5') and 4439/5799 (3'). SAT resistance cassette flipped out with maltose media.
CAY14854	Ssn6 CTD PO4 Ala satS	RB2180 digested with PspOMI and SacI and transformed into TF121. Integration checked with oligos 5683/8036 (5') and 4439/5799 (3'). SAT resistance cassette flipped out with maltose media.
CAY14855	Ssn6 CTD PO4 Ala satS	RB2180 digested with PspOMI and SacI and transformed into TF121. Integration checked with oligos 5683/8036 (5') and 4439/5799 (3'). SAT resistance cassette flipped out with maltose media.
CAY14856	Ssn6 CTD PO4 Asp satS	RB2181 digested with PspOMI and SacI and transformed into TF121. Integration checked with oligos 5683/8036 (5') and 4439/5799 (3'). SAT resistance cassette flipped out with maltose media.
CAY14857	Ssn6 CTD PO4 Asp satS	RB2181 digested with PspOMI and SacI and transformed into TF121. Integration checked with oligos 5683/8036 (5') and 4439/5799 (3'). SAT resistance cassette flipped out with maltose media.
CAY13555	Ssn6 full Neon satS	Long oligos 7113/7115 with Ssn6 homology used to amplify Neon-SAT cassette sequence from plasmid RB895. PCR product transformed into CAY11369. Integration checked with oligos 6350/5091 (5') and 4439/5684 (3'). SAT resistance cassette flipped out with maltose media.
CAY13556	Ssn6 full Neon satS	Long oligos 7113/7115 with Ssn6 homology used to amplify Neon-SAT cassette sequence from plasmid RB895. PCR product transformed into CAY11369. Integration checked with oligos 6350/5091 (5') and 4439/5684 (3'). SAT resistance cassette flipped out with maltose media.
CAY13557	Ssn6ΔN Neon satS	Long oligos 7113/7115 with Ssn6 homology used to amplify Neon-SAT cassette sequence from plasmid RB895. PCR product transformed into CAY11375. Integration checked with oligos 6350/5091 (5') and 4439/5684 (3'). SAT resistance cassette flipped out with maltose media.
CAY13558	Ssn6ΔN Neon satS	Long oligos 7113/7115 with Ssn6 homology used to amplify Neon-SAT cassette sequence from plasmid RB895. PCR product transformed into CAY11375. Integration checked with oligos 6350/5091 (5') and 4439/5684 (3'). SAT resistance cassette flipped out with maltose media.
CAY13559	Ssn6ΔM Neon satS	Long oligos 7113/7115 with Ssn6 homology used to amplify Neon-SAT cassette sequence from plasmid RB895. PCR product transformed into CAY11381. Integration checked with

		oligos 6350/5091 (5') and 4439/5684 (3'). SAT resistance cassette flipped out with maltose media.
CAY13560	Ssn6ΔM Neon satS	Long oligos 7113/7115 with Ssn6 homology used to amplify Neon-SAT cassette sequence from plasmid RB895. PCR product transformed into CAY11381. Integration checked with oligos 6350/5091 (5') and 4439/5684 (3'). SAT resistance cassette flipped out with maltose media.
CAY13561	Ssn6ΔNM Neon satS	Long oligos 7113/7115 with Ssn6 homology used to amplify Neon-SAT cassette sequence from plasmid RB895. PCR product transformed into CAY11387. Integration checked with oligos 6350/5091 (5') and 4439/5684 (3'). SAT resistance cassette flipped out with maltose media.
CAY13562	Ssn6ΔNM Neon satS	Long oligos 7113/7115 with Ssn6 homology used to amplify Neon-SAT cassette sequence from plasmid RB895. PCR product transformed into CAY11387. Integration checked with oligos 6350/5091 (5') and 4439/5684 (3'). SAT resistance cassette flipped out with maltose media.
CAY13563	Ssn6ΔT Neon satS	Long oligos 7113/7115 with Ssn6 homology used to amplify Neon-SAT cassette sequence from plasmid RB895. PCR product transformed into CAY13592. Integration checked with oligos 6350/5091 (5') and 4439/5684 (3'). SAT resistance cassette flipped out with maltose media.
CAY13564	Ssn6ΔT Neon satS	Long oligos 7113/7115 with Ssn6 homology used to amplify Neon-SAT cassette sequence from plasmid RB895. PCR product transformed into CAY13593. Integration checked with oligos 6350/5091 (5') and 4439/5684 (3'). SAT resistance cassette flipped out with maltose media.
CAY13565	Ssn6ΔC Neon satS	Long oligos 7114/7115 with Ssn6 homology used to amplify Neon-SAT cassette sequence from plasmid RB895. PCR product transformed into CAY13594. Integration checked with oligos 6455/5091 (5') and 4439/5684 (3'). SAT resistance cassette flipped out with maltose media.
CAY13566	Ssn6ΔC Neon satS	Long oligos 7114/7115 with Ssn6 homology used to amplify Neon-SAT cassette sequence from plasmid RB895. PCR product transformed into CAY13595. Integration checked with oligos 6455/5091 (5') and 4439/5684 (3'). SAT resistance cassette flipped out with maltose media.
